# A single chlamydial protein reshapes the plasma membrane and serves as recruiting platform for central endocytic effector proteins

**DOI:** 10.1038/s42003-023-04913-z

**Published:** 2023-05-13

**Authors:** Dominik Spona, Philipp T. Hanisch, Johannes H. Hegemann, Katja Mölleken

**Affiliations:** Institute for Functional Microbial Genomics, Heinrich-Heine-University, Düsseldorf, Germany

**Keywords:** Pathogens, Membrane lipids, Membrane curvature

## Abstract

Uptake of obligate intracellular bacterial pathogens into mammalian epithelial cells is critically dependent on modulation of the host’s endocytic machinery. It is an open question how the invading pathogens generate a membrane-bound vesicle appropriate to their size. This requires extensive deformation of the host plasma membrane itself by pathogen-derived membrane-binding proteins, accompanied by substantial F-actin-based forces to further expand and finally pinch off the vesicle. Here we show that upon adhesion to the host cell, the human pathogenic bacterium *Chlamydia pneumoniae* secretes the scaffolding effector protein CPn0677, which binds to the inner leaflet of the invaginating host’s PM, induces inwardly directed, negative membrane curvature, and forms a recruiting platform for the membrane-deforming BAR-domain containing proteins Pacsin and SNX9. In addition, while bound to the membrane, CPn0677 recruits monomeric G-actin, and its C-terminal region binds and activates N-WASP, which initiates branching actin polymerization via the Arp2/3 complex. Together, these membrane-bound processes enable the developing endocytic vesicle to engulf the infectious elementary body, while the associated actin network generates the forces required to reshape and detach the nascent vesicle from the PM. Thus, Cpn0677 (now renamed SemD) acts as recruiting platform for central components of the endocytic machinery during uptake of chlamydia.

## Abstract (web summary)

A scaffolding effector protein SemD secreted by Chlamydia pneumoniae acts as a recruiting platform for central components of the host endocytic machinery during uptake of the human pathogenic bacterium.

## Introduction

*Chlamydia pneumoniae* (*Cpn*) primarily infects the upper and lower respiratory tract, causing acute sinusitis, bronchitis, and pneumonia^1^. In addition, the pathogen is associated with chronic diseases of the respiratory tract and other organs, including asthma, lung cancer, Alzheimer’s disease, and multiple sclerosis^1–3^. The infection process is initiated by the interaction of adhesins located on the surface of the infectious ‘elementary body’ (EB) with receptors on the plasma membrane (PM) of the host cell. Adhesion triggers the formation of an atypical endocytic vesicle, in which the EB is encapsulated and internalized within the cell. Since EBs are larger (300–400 nm) than normal endocytic vesicles, successful uptake requires extensive reshaping and remodeling of the nascent vesicular membrane. The necessary reorganization is mediated by chlamydial effector proteins, which are secreted into the host cell upon adhesion/internalization. Among the central targets of these effectors are host proteins that are involved in (i) binding, modulating and deforming the PM, and (ii) modifying actin dynamics^4,5^. Previously, we were able to identify SemC (CPn0678) the first described early effector, which is able to deform the PM in order to enhance the process of entry of *Cpn*^6^. During endocytosis membrane modulations and actin dynamics are intimately intertwined. The actin cytoskeleton is tightly linked to the invaginating membrane, and generates forces that enlarge the developing vesicle and eventually detach it from the PM^7^. Targeting of the actin-modulating machinery is therefore utilized by many intracellular bacterial pathogens. Many of these, such as *Salmonella* species, have evolved multiple effectors which intervene in actin polymerization either by binding to actin itself or by interacting with central regulatory components such as the neural Wiskott–Aldrich syndrome protein (N-WASP), Arp2/3 or their upstream and downstream signaling pathways (actin effectors)^8^. In *Chlamydia* species, the effectors TarP and TmeA are examples of this strategy^9,10^. Moreover, to induce the required membrane curvature during entry several intracellular pathogens also secrete effector proteins that target a particular class of host membrane-deforming proteins known as bin-amphiphysin-rvs (BAR) domain proteins^6,11,12^. Their banana-shaped BAR domains bind to membranes and, depending on the type of BAR domain involved, induce positive or negative membrane curvature with the aid of the actin-modulating machinery^13–15^. The major BAR proteins in endocytosis are sorting nexin 9 (SNX9) and Pacsin (also known as syndapin). SNX9 binds to the neck of the vesicle, and induces vesicle closure by recruiting the GTPase dynamin, which activates the actin modulator N-WASP. This in turn leads to Arp2/3-mediated actin polymerization, and in concert with the membrane-deforming capacity of SNX9, results in constriction of the PM until the vesicle is released into the cytosol^16^. Pacsin on the other hand, binds as an oligomer to the edge of the developing vesicle, which initially becomes U-shaped and is then expanded into a larger omega-like structure^7^. Pacsin generates narrow membrane tubules with the aid of a specialized F-BAR domain^17^. Furthermore, the protein interacts with N-WASP through SH3 and proline-rich domains. This latter interaction results in actin polymerization at Pacsin-rich loci during endocytosis, which provides the mechanical forces necessary to reshape the membrane during vesicle formation^18,19^.

Here, we demonstrate that the early effector protein CPn0677 connects two essential mechanisms of pathogen-controlled endocytosis—membrane deformation and actin dynamics by acting as a scaffold. Upon secretion, CPn0677 binds to, and deforms the invaginating membrane, and recruits the host BAR proteins SNX9 and Pacsin, which further remodel the invaginating membrane. Furthermore, CPn0677 not only recruits and activates N-WASP, it also contributes G-actin molecules to subsequent actin polymerization. Together with N-WASP, this enhances local actin polymerization, which drives the degree of membrane deformation necessary for successful *Cpn* endocytosis.

## Results

### Secreted CPn0677 exerts strong membrane-deforming forces

**Fig. 1:**
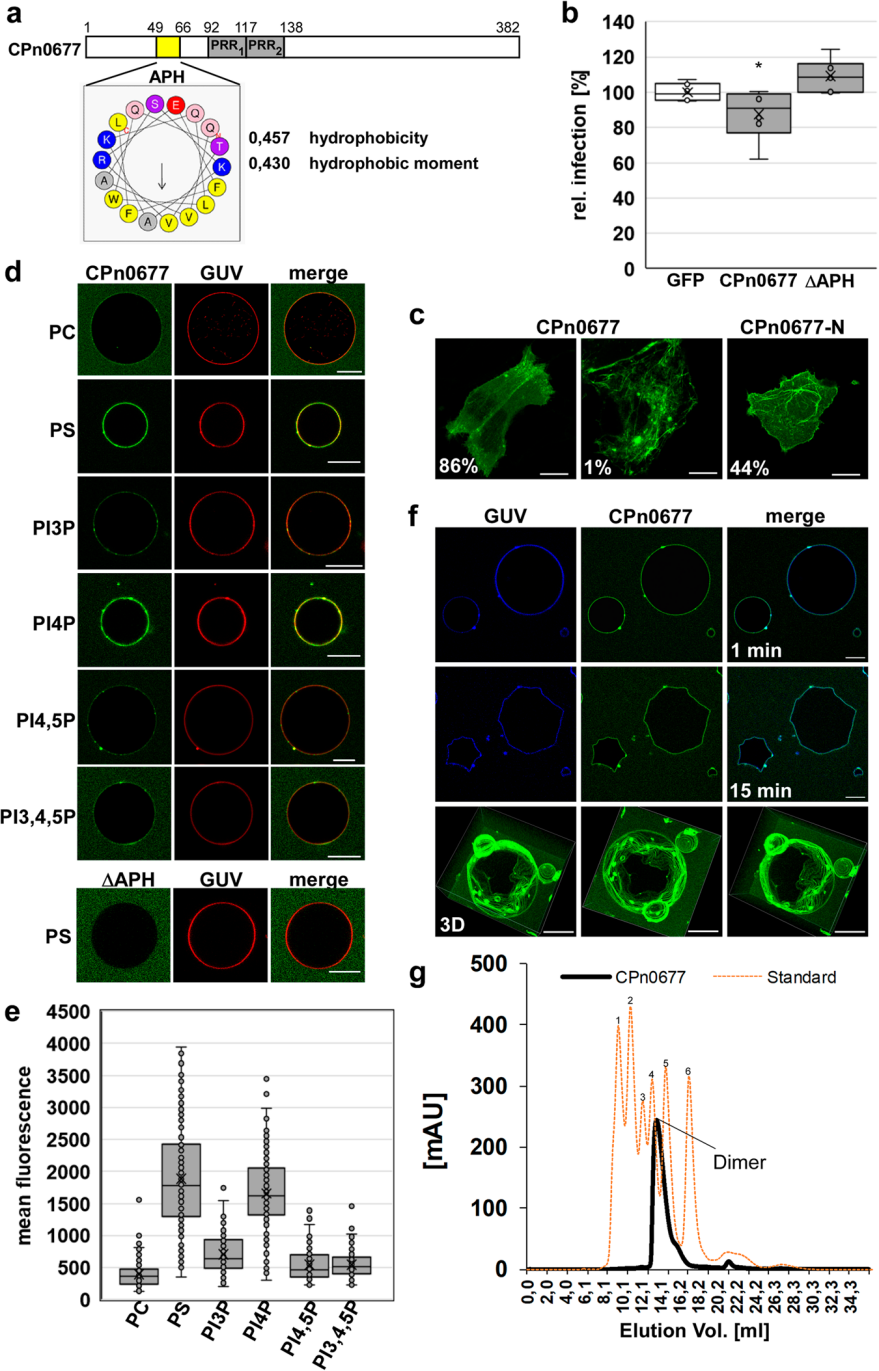
The C-terminal segment of CPn0677 mediates membrane binding. **a** Schematic representation of the domain structure of CPn0677. The position of the amphipathic helical region (APH, aa 49-66) is highlighted in yellow and was analyzed for hydrophobicity, hydrophobic moment and amino-acid composition using HeliQuest (heliquest.ipmc.cnrs.fr). Gray boxes represent proline-rich repeats (PRRs, aa 92–116; aa 117–138). **b** Quantification of inclusion formation in HEp-2 cells expressing either GFP, CPn0677 or CPn0677ΔAPH fused to GFP challenged with *Cpn* EBs (MOI1) 24 h post transfection. Inclusion numbers were quantified 48 hpi by staining the inclusion with an anti-LPS antibody and an anti-mouse antibody coupled to Alexa594. Inclusion numbers were normalized to those detected in GFP expressing control cells. ( ± SD, *n* = 3 biologically independent experiments) *P* = *≤ 0.05. **c** Confocal images of HEp-2 cells expressing CPn0677 or CPn0677-N fused to GFP. Bar: 10 µm. **d** Binding of FITC-labeled recombinant CPn0677 variants to GUVs containing the indicated lipids stained with Texas red and of GUVs incubated with CPn0677 and its N-terminal deletion derivative ΔAPH. Bar 10 µm. **e** Quantification of protein binding to GUVs containing the indicated lipids. Data are expressed as the mean fluorescence ( ± SD, *n* = 3 biologically independent experiments) of 50 GUVs shown in (**d**). **f** Confocal images of PS-GUVs labeled with Marina Blue™ and incubated with FITC-labeled CPn0677 (1 µM) for the indicated times (see also Supplementary Movie 1). Note the CPn0677-mediated membrane deformation at the 15-min timepoint. The bottom row shows 3D z-stack images taken from various angles at 15 min. Bar 10 µm. **g** SEC of 5.2 mg CPn0677 dissolved in PBS and separated on a superdex 200 increase 10/300 GL column depicted as solid black lane. The SEC profile of the molecular weights of the standard proteins is shown in orange dashed line (^1^Thyroglobulin 669 kDa, ^2^Apoferritin 480 kDa, ^3^β-Amylase 200 kDa, ^4^Alcohol Dehydrogenase 150 kDa, ^5^Albumin 66 kDa, ^6^Carbonic Anhydrase 29 kDa). mAU absorbance units. (*n* = 2 biologically independent experiments).

**Fig. 2:**
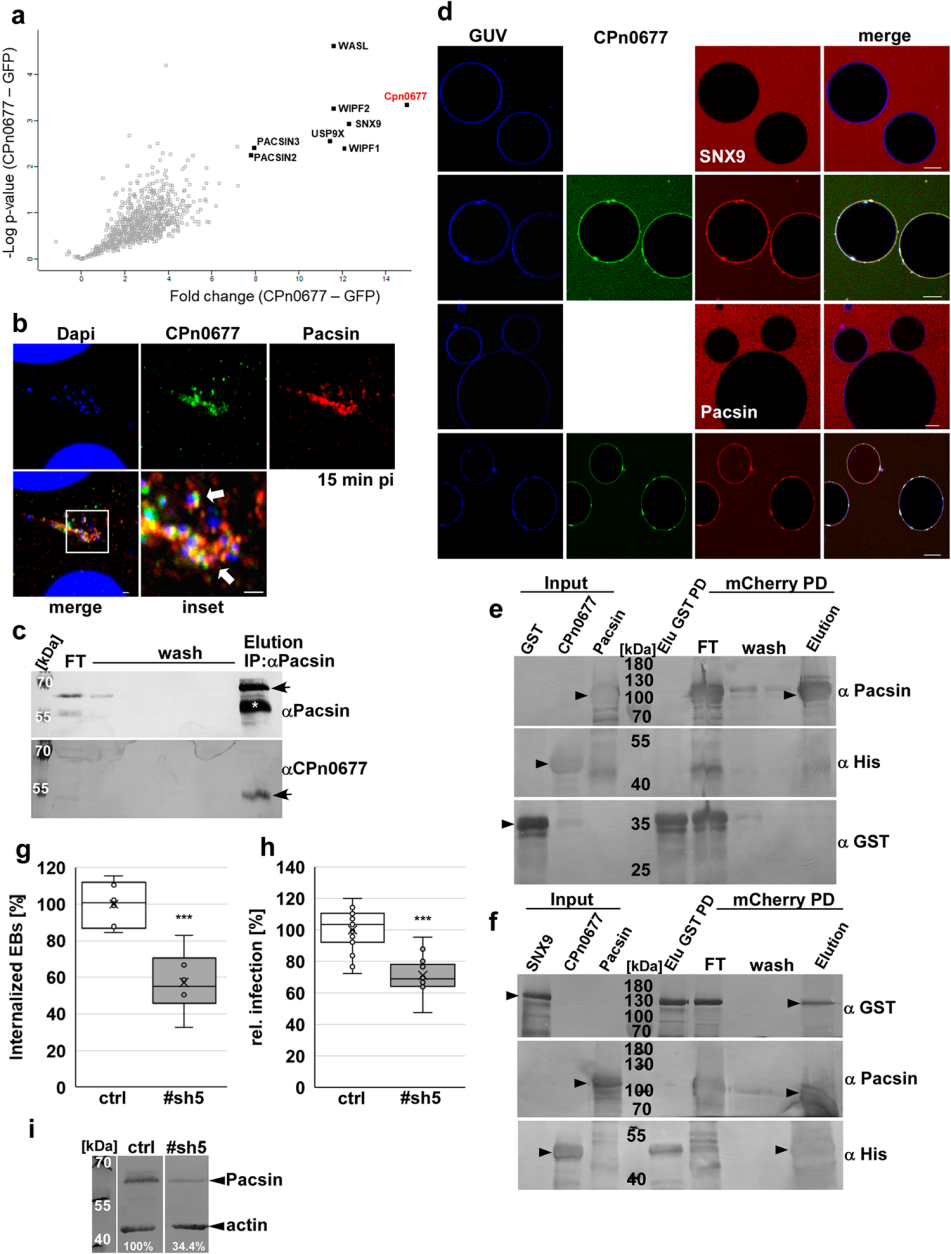
CPn0677 interaction with the human BAR protein Pacsin is essential for Cpn endocytosis. **a** Volcano plot of proteins (Uniprot nomenclature) detected following affinity purification with CPn0677-GFP as bait (*n* = 3) and subsequent analysis by quantitative mass spectrometry. Proteins detected in the highest amounts relative to GFP-only controls (*n* = 3 biologically independent experiments) are represented by black squares; all others are marked as open gray squares. The fold change (CPn0677-GFP minus GFP) is plotted against the difference in mean values of log2 label-free quantitation (LFQ) intensities (CPn0677-GFP minus GFP). **b** Colocalization of Pacsin (stained with anti-Pacsin and anti-mouse Alexa594) and CPn0677 (stained with anti-CPn0677 and anti-rat Alexa488) at bacterial entry sites at 15 min pi. *C. pneumoniae* EBs were stained with DAPI. The inset shows the region outlined by the white frame. White arrowheads show colocalization Bar = 10 µm, inset = 1 µm. **c** Co-immunoprecipitation of Pacsin and Cpn0677 from HEp-2 cells infected with *C. pneumoniae* EBs (MOI 100). Cell lysates were incubated with µMACS protein G microbeads coupled to antibodies directed against Pacsin. Eluates were then fractionated by SDS/PAGE and incubated with anti-Pacsin and anti-CPn0677 antibodies. The white asterisk marks the heavy chain of the antibody, the black arrows indicate the specifically labeled Pacsin and CPn0677 bands, respectively. **d** Confocal images of PS-GUVs labeled with Marina Blue™ that were incubated and imaged for 10 min with either 1 µM NHS-rhodamine-labeled SNX9 or Pacsin alone, followed by the addition of FITC-labeled 1 µM CPn0677. Images shown here were taken 10 min after the addition of CPn0677. Bar = 10 µm. **e**, **f** Consecutive GST and mCherry pulldowns in which first either GST (**e**) or SNX9-GST (**f**) was incubated with CPn0677_10His_ and purified via GST agarose. Elution fractions were than incubated with Pacsin-mCherry and purified via mCherry Trap® agarose. Fractions from both pulldowns were separated by SDS/PAGE and probed with anti-GST, anti-Pacsin and anti-His antibodies. Black arrowheads indicate full-length protein in input and final elution fractions. **g**, **h** *C. pneumoniae* internalization and infection of HEp-2 cells that had been depleted of Pacsin by expression of stably integrated shRNA plasmids. A non-targeting shRNA served as control. In both panels, data are based on the examination of 40 visual fields and presented are means ( ± SD, *n* = 3 biologically independent experiments). *P* = *** ≤ 0.001. **g** Quantification of internalization of EBs into cells at 2 hpi. **h** Quantification of infection of cells at 48 hpi based on the number of inclusions in HEp-2 cells. **i** Immunoblot analysis of lysates of HEp-2 cells stably expressing either a control shRNA or a shRNA directed against Pacsin (#sh5), Lysates were fractionated by SDS/PAGE and probed with antibodies against Pacsin. Actin served as the loading control. Signal intensity was quantified with ImageJ and expressed as a percentage of the actin signal.

**Fig. 3:**
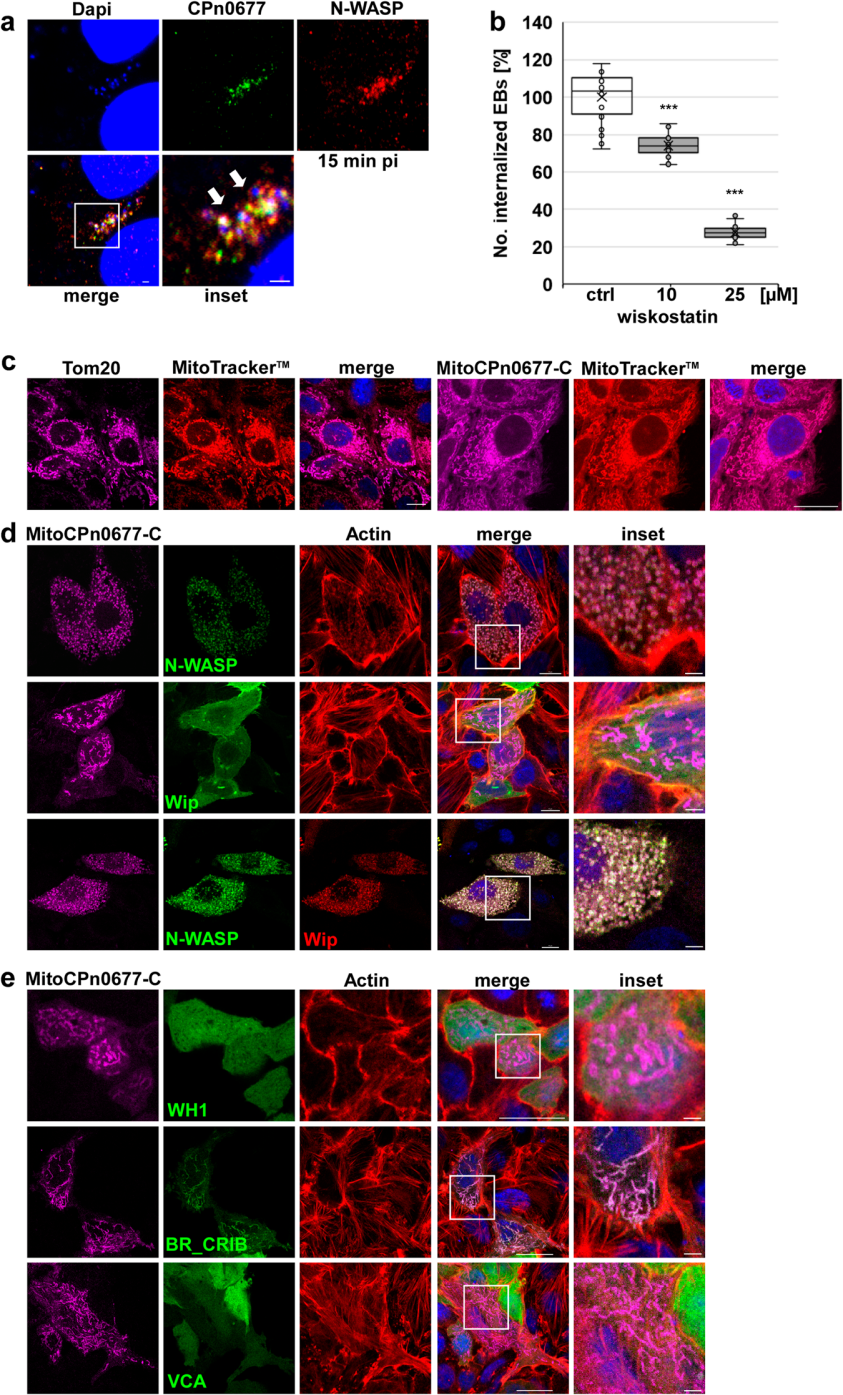
CPn0677 recruits N-WASP to bacterial entry sites and activates it. **a** Colocalization of N-WASP (stained with anti-N-WASP and anti-mouse Alexa594 antibodies) and CPn0677 (stained with anti-CPn0677 and anti-rat Alexa488 antibodies) at bacterial entry sites at 15 min pi. *C. pneumoniae* EBs were stained with DAPI. The inset shows region outlined by the white frame. White arrowheads show areas of colocalization. Bar = 10 µm, insets 1 = µm. **b** Quantification of uptake of EBs into HEp-2 cells pretreated for 5 min with either DMSO or wiskostatin (10 and 25 µM) and washed three times with PBS prior to infection. At 2 hpi cells were fixed, and external EBs were stained using an anti-LPS antibody in combination with anti-mouse Alexa488. All EBs were stained with DAPI. External and internalized EBs were quantified based on the examination of 40 visual fields. Data are represented as means ± SD (*n* = 4 biologically independent experiments). *P* value: ***≤0.001. **c** HEp-2 cells expressing either Tom20 or MitoCPn0677-C fused with SNAP were stained with MitoTracker™Red. DNA was visualized with DAPI. Bar = 10 µm. **d**, **e** Confocal images of the mitochondrion-targeted MitoCPn0677-C, co-expressed with the indicated N-WASP variants and the N-WASP interactor Wip. Insets show regions outlined by the white frames. **d** HEp-2 cells co-expressing MitoCPn0677-C fused to SNAP with wild-type GFP-N-WASP or GFP-Wip. **e** HEp-2 cells co-expressing MitoCPn0677-C and different N-WASP subdomains (depicted in Supplementary Fig. S2b) fused to GFP. Cells were fixed and F-actin was stained with rhodamine-phalloidin. DNA is visualized using DAPI. Bar = 10 µm, insets = 5 µm.

The *Cpn*-specific protein CPn0677, homolog to SemC, (Fig. 1a) is secreted during the first 15 min of infection (Figs. 2b and 3a) via the Type 3 secretion system (T3SS)^6^. Structural predictions indicate that it is made up of an N-terminal amphipathic α-helix (APH) with a significantly hydrophobic character suggesting that it interacts with membranes, and two centrally located proline-rich sequences (PRR1 and PRR2) (Fig. 1a). Ectopic expression of a full-length CPn0677-GFP fusion in human cells revealed a generally cytosolic distribution, with enrichment at the plasma membrane (PM) and in short tubule- and patch-like structures being detected in 86% of the cells examined. In 1% of expressing cells we observed extensive membrane tubules emanating from the PM into the cytosol (Fig. 1c). However, when an N-terminal fragment (CPn0677-N, aa 1–138, Supplementary Fig. S2b) was expressed, 44% of the cells exhibited the membrane localization and tubulation phenotype (Fig. 1c). These observations suggested that the APH has a strong membrane-binding and deforming capacity, whereas the C-terminus seems to contain further protein domains involved in other functions as membrane interaction. Secretion of CPn0677 is essential for the *Cpn* infection, as in cells ectopically expressing the protein and then challenged with an infection, a 22% reduction in inclusion formation was observed (Fig. 1b). Thus, intracellular presence of the protein negatively influences the infection process. This is due to the membrane-binding capacity of the protein as expression of an ΔAPH (deletion of the APH in full-length protein) variant could not influence a subsequent infection (Fig. 1b). To analyze the membrane-binding potential of CPn0677 further, we used giant unilaminar vesicles (GUVs) consisting of various phospholipids. When FITC-labeled CPn0677 was incubated with GUVs of distinctly different lipid composition, we found that the protein bound to most of those tested, but exhibited a strong preference for PI4P and PS (Fig. 1d, e), both of which are located in the inner leaflet of the PM. While PS is a major component of the PM, PI4P is rather short-lived there and resides predominantly in Golgi membranes^20,21^. Deletion of the APH resulted in the complete loss of binding to GUVs (Fig. 1d), indicating that this segment indeed represents the lipid-binding domain of the protein. Surprisingly, when we analyzed CPn0677 binding to PS-containing GUVs in more detail, we noted that, over time, CPn0677 is capable of stably deforming the typically sphere-shaped GUVs into characteristically angular structures (15 min; Fig. 1f and Supplementary Movie 1), apparently by exerting an inwardly directed force. This very peculiar phenotype is probably due to oligomerization of membrane-bound CPn0677. Indeed, size-exclusion chromatography (SEC) of recombinant CPn0677 protein revealed, that already in solution without any membrane present, the protein was able to form dimers (Fig. 1g). Thus, we speculate that upon membrane binding, the protein may form larger oligomers that due to their arrangement on the membrane lead to the observed membrane-deforming forces.

### The CPn0677 interactome uncovers central endocytotic proteins

In order to gain insight into the function of CPn0677 during *Cpn* internalization, we used a biochemical approach to identify the protein’s interactome. The CPn0677-GFP fusion protein ectopically expressed in HEp-2 cells was isolated and affinity-purified, and the human proteins that bound to it were identified by mass spectrometry. Intriguingly, all of the significantly enriched human proteins that interacted with CPn0677 (but not with the GFP control) are implicated in endocytic processes in eukaryotic cells (Fig. 2a). Among them we identified two membrane-associated, BAR-domain-containing proteins (SNX9 and Pacsins 2/3, here we analyzed Pacsin2 and refer to it as Pacsin), which are important for generating positive and negative membrane curvature during formation (Pacsin) and scission (SNX9) of endocytic vesicles^15^. Both proteins harbor SH3 domains, which are known to interact with proline-rich domains in various other components of the endocytic machinery^14^. Furthermore, N-WASP and its core interacting protein Wip (WASP-interacting protein, WIPF1/2), which are central modulators of actin dynamics, were among the top hits in the CPn0677 interactome (Fig. 2a). Active N-WASP mediates actin polymerization in an Arp2/3-dependent manner, and this is an essential step in endocytosis^7,22,23^. In addition to these classical endocytosis effectors, we found USP9X, a large de-ubiquitinating enzyme that regulates the ubiquitination status of the receptor for an epidermal growth factor (EGFR) during its internalization and trafficking^24^. EGFR is activated by the chlamydial adhesin and invasin Pmp21, and is the primary receptor for *Cpn* on host cells^25,26^. These findings indicate that secreted CPn0677 binds to the PM during the endocytosis of infectious EBs and can interact with various host proteins that are involved in the regulation of the endocytic process, including membrane-reshaping proteins and modulators of actin dynamics.

We therefore tested whether the identified proteins directly or indirectly interact with CPn0677 during *Cpn* uptake. First, we focused on key players in endocytosis—SNX9, Pacsin, and N-WASP. We found that endogenous Pacsin colocalizes with secreted CPn0677 surrounding invading EBs at 15 min post infection (pi) (Fig. 2b). This is also true for SNX9, which colocalizes with EBs at bacterial entry sites (Supplementary Fig. S1a). In addition, immunoprecipitation experiments showed that these endogenous BAR proteins were associated with CPn0677 at 15 min pi, and thus interact with the secreted bacterial effector protein (Fig. 2c; SNX9 data in Supplementary Fig. S1b), indicating that their colocalization is the consequence of recruitment and direct binding. To confirm that the interaction of CPn0677 with Pacsin/SNX9 is indeed direct, we performed pulldown assays with recombinant CPn0677-His, GST-Pacsin and GST-SNX9 proteins (Supplementary Fig. S1d, e), and verified that full-length CPn0677-His bound to GST-SNX9. Moreover, deletion of either the SH3 domain in SNX9 (ΔSH3, aa 1–60) or PRR1 in CPn0677 (ΔP1, aa 92–116) effectively prevented the interaction (Fig.S1d). PRR1 is 92% identical to PRR1 of SemC, which also uses the same sequence for interaction with SNX9^6^. Interaction of Pacsin with CPn0677 appears to be independent of the SH3 and PRR domains, as GST-PacsinΔSH3 (aa 1–428) still interacted with wild-type CPn0677 and its deletion variants CPn0677ΔPRR1 and CPn0677ΔPRR1 + 2 (aa 92–138) (Supplementary Fig. S1e). However, using CPn0677-C4 (aa 218–382, Supplementary Fig. S2b) we were able show that the binding domain for Pacsin must be located within the last 160 amino acids of CPn0677 (Supplementary Fig. S1e). These results show that the presence of SNX9 and Pacsin among the CPn0677 interactome reflects their ability to interact directly with the chlamydial protein. This suggests that CPn0677 recruits both BAR proteins to the PM at bacterial entry sites. Further proof came from experiments on synthetic membrane vesicles (Fig. 2d). When incubated with either labeled recombinant SNX9 or Pacsin alone, GUVs failed to bind to the either protein. Intriguingly, upon the addition of recombinant CPn0677 to these samples, rapid binding of Cpn0677 to the GUV membrane, and concomitant recruitment of the two BAR proteins, was observed (Fig. 2d). In a biochemical in vitro experiment, we could show that CPn0677 can engage in a trimeric complex with both BAR proteins probably due to its own oligomerization. When we purified a binary complex of CPn0677-His and SNX9-GST or using GST agarose and then added Pacsin-mCherry to this complex, we found all three proteins present in the elution fractions of a subsequent purification via mCherry Trap® agarose (Fig. 2f). GST alone served as a control (Fig. 2e) as well as experiments in which we detected no binding of SNX9 and Pacsin to one another (Supplementary Fig. S1f). These data imply that membrane-bound, oligomeric CPn0677 can recruit SNX9 and Pacsin simultaneously to remodel the PM.

Pacsin functions in clathrin-mediated endocytosis (CME) and caveolae formation, as well as in endosomal and vesicle trafficking. The protein combines a membrane remodeling activity mediated by its specialized F-BAR domain, which induces high membrane curvature, with the recruitment of various key players in endocytosis, such as dynamin, synaptogenin and N-WASP, via SH3-PRR interactions^18^. Indeed, the integration of N-WASP-mediated actin polymerization during endocytosis is an essential function of Pacsin^27^. Thus, we wondered whether Pacsin is also indispensable for the endocytosis of infectious *Cpn* EBs. To this end, we generated various stable cell lines expressing shRNAs directed against Pacsin, and observed an approximately threefold reduction in the level of the protein in line #sh5 (Fig. 2i). Infection of these cells with *Cpn* EBs resulted in a 30% reduction in internalized EBs and a 29% reduction in infection, respectively, relative to controls (Fig. 2g, h), proving that Pacsin is essential for the *Cpn* infection.

### CPn0677 interacts with N-WASP independent of other endocytotic proteins

The previous data support a direct interaction of membrane-bound CPn0677 with both SNX9 and Pacsin, each of which is known to recruit N-WASP^27,16^. Since our CPn0677 interactome analysis had identified N-WASP in addition to SNX9 and Pacsin, we asked whether N-WASP is a direct or indirect interaction partner of CPn0677. Microscopical analysis of the infection process at 15 min pi revealed a strong enrichment of endogenous N-WASP around secreted CPn0677 at bacterial entry sites (Fig. 3a). The interaction of N-WASP and CPn0677 was subsequently confirmed by co-immunoprecipitation of the two proteins at this early stage of infection (Supplementary Fig. S2a). To directly test whether N-WASP plays a role in EB internalization, HEp-2 cells were pretreated with the N-WASP inhibitor wiskostatin, which traps the protein in an inactive state^28^, prior to infection. In these cells, a dose-dependent reduction in internalization of EBs, ranging from 27% at 10 mM to 73% at 25 mM (Fig. 3b), was observed, showing that N-WASP localizes to internalizing EBs and is essential for the endocytosis of *Cpn*.

This finding raised the question whether N-WASP is recruited via Pacsin and/or SNX9 or directly by CPn0677. To answer it, we performed colocalization studies on the various proteins ectopically expressed in human cells, and found that CPn0677-GFP localizes to the cytoplasm, and to patch-like structures to which all of the human proteins in question localize (Supplementary Fig. S2c). These CPn0677-containing patches are highly enriched in actin, as revealed by staining with phalloidin (Supplementary Fig. S2c), indicating that overexpression of the chlamydial effector recruits its interactors into structures that are engaged in actin dynamics. Next, we tested the truncated CP0677-GFP variant (CPn0677-N, aa 1–138) that includes the APH and the PRRs (Supplementary Fig. S2b) in combination with either mCherry-Pacsin, mCherry-SNX9 or mCherry-N-WASP to elucidate the inter-dependencies required for recruitment. As previously described, CPn0677-N induces a pronounced membrane tubulation phenotype, while SNX9 and—surprisingly—Pacsin colocalize with CPn0677-N at the PM and on the membrane tubules (Supplementary Fig. S2d). Localization of SNX9 was expected, owing to its affinity for PRR1, but Pacsin also reveals an affinity for the PRRs in Cpn0677, upon deletion of the C-terminal segment of the latter (Supplementary Fig. S2d). Interestingly, we observed no colocalization of CPn0677-N with N-WASP (Supplementary Fig. S2d). This implies that colocalization of CPn0677 with N-WASP is independent of both Pacsin and SNX9 binding, and must therefore be mediated by the C-terminus of CPn0677. Indeed, expression of CPn0677-C (aa 126–382, Supplementary Fig. S2b) lacking the membrane-binding domain resulted in a diffuse cytoplasmic localization and in patch-like structures that localized with N-WASP (Supplementary Fig. S2d).

### CPn0677 directly binds and activates human N-WASP

To study the CPn0677/ N-WASP interaction in more detail, and identify interaction domains in protein variants lacking their endogenous membrane-binding capacity, we decided to use the mitochondrial outer membrane as an artificial in vivo interaction platform. We targeted the N-WASP-binding C-terminal region of CPn0677 (aa 126–382) to the mitochondrial outer membrane by fusing it to a SNAP-tag and the N-terminal Tom20 signal sequence (MitoCPn0677-C). Mitochondria are dynamic organelles that form complex networks in the cell, and the balance between mitochondrial fission and fusion is regulated by actin polymerization, which is mediated by mitochondria-specific actin-binding and nucleating proteins^29^. Following transient expression in human cells, MitoCPn0677-C localizes to mitochondria, which retain an apparently wild-type morphology in comparison to the Tom20 control (Fig. 3c). Intriguingly, co-expression of MitoCPn0677-C with N-WASP resulted in a drastic change in mitochondrial morphology (Fig. 3d), as the organelle disintegrates into small rings covered with CPn0677-C, N-WASP, and F-actin (Fig. 3d). This indicates that the C-terminus of CPn0677 recruits N-WASP to the mitochondrial outer membrane, which results in local actin polymerization events that lead to fission of the organelles. This effect is N-WASP-dependent, as co-expression of MitoCPn0677-C with the N-WASP-interacting protein Wip (also found in the CPn0677 interactome) did not result in colocalization of the two proteins, and the mitochondria were not morphologically altered (Fig. 3d). This suggests that Wip is a bystander co-isolated as an CPn0677 interaction partner owing to its binding to N-WASP. Indeed, co-expression of Wip and N-WASP together with MitoCPn0677-C again resulted in the disintegration of mitochondria into rings, which are now decorated with all three proteins. This suggests that CPn0677 recruits and binds N-WASP, which in turn binds to Wip.

To identify the Cpn0677-interacting domain within N-WASP, we dissected the protein into sub-fragments corresponding to its major protein domains (WH1, BR_CRIB and VCA; see Supplementary Fig. S2b)^23^. Co-expression of the individual N-WASP domains with the mitochondrially targeted CPn0677-C revealed that neither the WH1 domain (which is important for Wip interaction) nor the VCA domain (required for G-actin and Arp2/3 binding) interacts with CPn0677 (Fig. 3e). However, the BR_CRIB domain, which binds to small GTPases and is essential for their intrinsic autoinhibition, binds to CPn0677—without changing mitochondrial morphology (Fig. 3d). These results imply that, while BR_CRIB interacts with Cpn0677, the other N-WASP domains are required for actin polymerization, and thus for changes in mitochondrial morphology.

N-WASP function during actin polymerization is tightly regulated, and requires interactions with many different proteins at specific locations and times. Autoinhibition of N-WASP is mediated by an intramolecular interaction of the CRIB domain with the central C-terminal hydrophobic region (C-region) of the VCA domain^30^. During endocytosis, N-WASP is activated by the binding of Cdc42 to the CRIB domain, which “opens up” the molecule so that it can interact with other proteins involved in regulating actin polymerization at the membrane^31^. The data presented here suggest that, during *Cpn* uptake, secreted CPn0677 binds to the PM and recruits N-WASP to the membrane below the attached chlamydial EBs via interaction with the BR_CRIB domain, thus initiating local actin dynamics. Interestingly, bioinformatic analyses of the C-terminal portion of Cpn0677 has revealed the presence of two potential CRIB-interacting motifs, which are common to N-WASP-interacting proteins (Nck^32^), (EspF and EspF(U)^33^), (TmeA^10^) (Supplementary Fig. S2b). Indeed, N-WASP interaction studies using recombinant BR_CRIB and diverse CPn0677 deletion variants showed that only wild-type CPn0677 and the C4 variant harboring both of the predicted motifs, but not CPn0677-N3, bind to BR_CRIB (Supplementary Fig. S2e). This provides evidence that CPn0677 binds to N-WASP via those two C-terminal binding motifs. Importantly, these data show that CPn0677 can bind directly to N-WASP without the aid of Pacsin or SNX9. To test whether CPn0677-mediated N-WASP recruitment also occurs in a cell-free system, a recombinant mini-WASP comprised of the BR_CRIB and VCA domains connected by a glycine-serine linker was produced (Supplementary Fig. S2b). Indeed, mini-WASP does not bind to GUV membranes (Supplementary Fig. S2f). However, upon the addition of CPn0677, immediate recruitment of mini-WASP to CP0677 sites on the surface of the GUV is observed (Supplementary Fig. S2f). In conclusion, CPn0677 is itself able to recruit N-WASP to the membrane directly. This suggests that direct recruitment of N-WASP also occurs at chlamydial entry sites, where N-WASP-induced actin polymerization is required for the uptake of the EB.

### CPn0677 is a multifactorial protein hub, directly binding G-actin to initiate polymerization

**Fig. 4:**
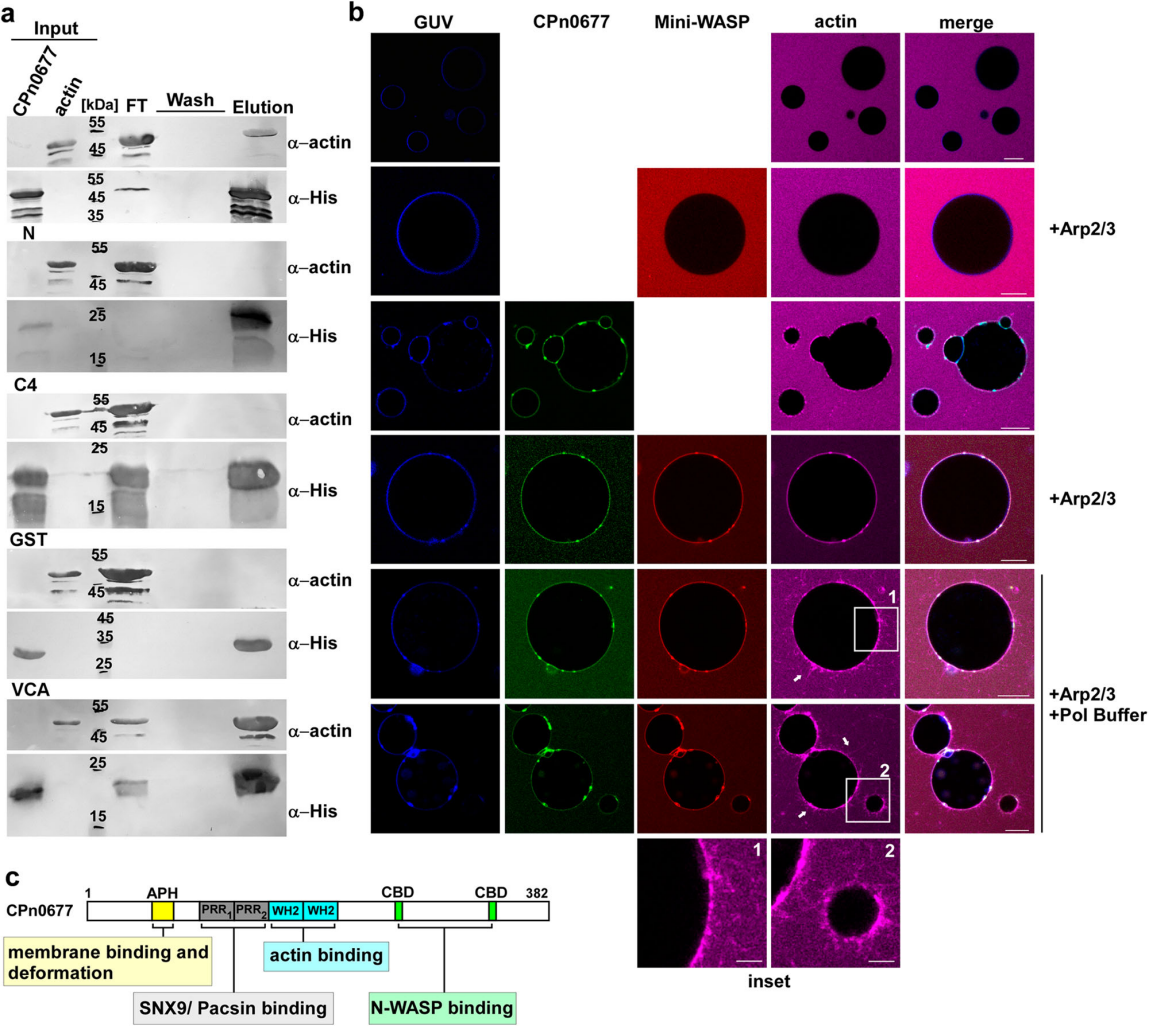
G-actin-bound CPn0677 recruits and activates human N-WASP to polymerize actin filaments in PS-containing GUVs. **a** Pulldown experiments using monomeric G-actin (100 µg) in combination with His-tagged CPn0677, CPn0677-N, or CPn0677-C4 (100 µg). The His-tagged VCA domain (N-WASP) and GST served as positive and negative controls, respectively. Input, flow through (FT), wash and elution samples obtained from His-pulldowns were fractionated by SDS/PAGE and probed with anti-actin and anti-His antibodies. **b** Confocal images of PS-containing GUVs labeled with Marina Blue™, and incubated and imaged for 10–15 min after addition of the various labeled and unlabeled proteins. The top two rows show GUVs that had been incubated with 3 µM G-actin Atto647 alone or in combination with 3 µM NHS-rhodamine-labeled mini-WASP and unlabeled Arp2/3 complex (100 nM). For next two rows 3 µM FITC-labeled CPn0677 was added to the mixture. In the last two last rows G-actin, CPn0677, Mini-WASP and Arp2/3 were added to the GUVs before actin polymerization was started by addition of 1 x actin polymerization buffer. Actin polymerization on the GUV membrane was imaged for 15 min. Bar = 10 µm. **c** Schematic representation of the multifactorial interactions of Cpn0677 with different, central endocytic host proteins analyzed in this report.

Our findings thus indicate that CPn0677 recruits N-WASP and activates it for subsequent actin polymerization. Active N-WASP will dimerize, and each protein will bind two G-actin molecules via its two WH2 domains (WASP-homology domain 2) in the Verprolin homology domain^34^. Interestingly, further examination of the sequence of CPn0677 identified two predicted WH2 domains in the center of CPn0677 (Supplementary Fig. S2b). Thus, we tested whether CPn0677 was able to bind G-actin in vitro. Indeed, in a pulldown with recombinant proteins, the wild-type protein binds monomeric actin (Fig. 4a). This interaction was confirmed by testing CPn0677-N and CPn0677-C4 variants, both lacking the predicted WH2 domains, which showed no actin binding, As expected, the VCA domain of N-WASP, harboring the two WH2 domains, also bound G-actin, while recombinant GST showed no binding to G-actin (Fig. 4a). Thus, the data show that the two predicted WH2 domains within CPn0677 are indeed capable of binding G actin.

We next asked whether G-actin also binds to membrane-bound CPn0677. We incubated GUVs with fluorescently labeled G-actin alone or in combination with Mini-WASP (which contains two G-actin-binding domains) and Arp2/3, and detected no recruitment of G-actin to the synthetic membrane. Remarkably, however, upon the addition of labeled CPn0677 to samples containing G-actin alone, we observed immediate recruitment of the actin monomers to the GUV membrane (Fig. 4b and Supplementary Movie 2). When we added CPn0677 to samples containing G actin and Mini-WASP, the GUV membrane was stained for all three proteins, with G-actin being enriched (Fig. 4b and Supplementary Movie 3). These data strongly indicate that CPn0677 binds to membranes and recruits both the actin-modulating protein Mini-WASP and monomeric actin. The G-actin concentration in the vicinity of the membrane is maximized because both mini-WASP and CPn0677 contribute G-actin molecules bound to their WH2 domains.

Finally, we repeated this experiment and initiated actin polymerization by increasing the concentrations of Mg^2+^ and KCl. Remarkably, we observed actin polymerization not only in our solution where all unbound proteins due to high concentration can form actin filament but most astoundingly, we see these actin filaments emerging from the surface of the GUVs (Fig. 4b, insets and Supplementary Movie 4). Thus, CPn0677 binding to the GUV membrane provides a platform for the actin polymerization machinery. Taken together, our findings reveal that CPn0677 not only binds to, and deforms membranes, it also recruits and activates N-WASP at the membrane, which leads to actin polymerization. Owing to its own actin-binding capacity, polymerization is further enhanced. These findings prompted us to rename CPn0677 as a “secreted effector of membrane and actin dynamics” (SemD).

## Discussion

**Fig. 5:**
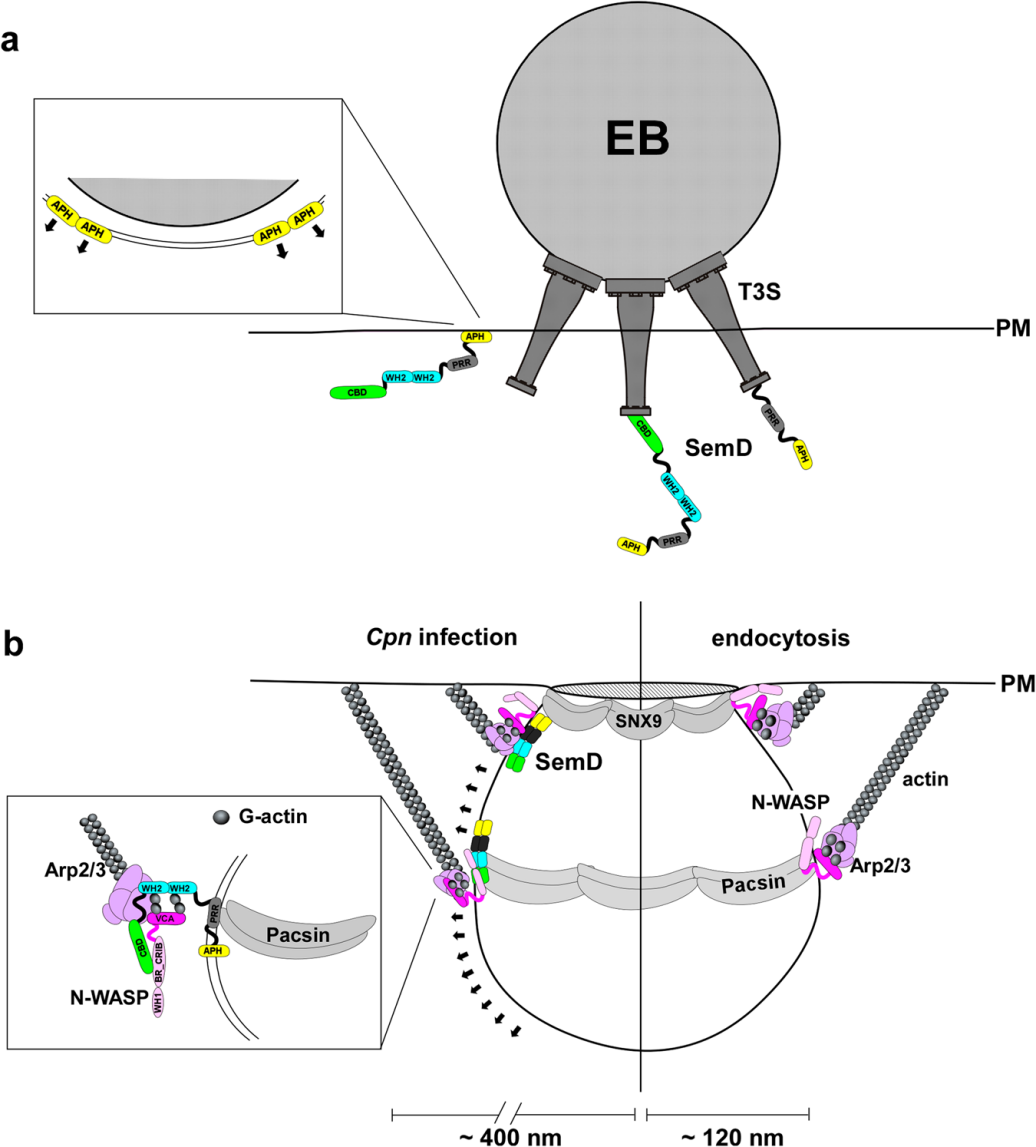
Model: CPn0677 bound to and reshaping the plasma membrane serves as recruiting platform during Cpn endocytosis. **a** During adhesion SemD (CPn0677) is secreted into the host cell cytosol via the Type 3 secretion system (T3S). Inside the cell, SemD dimers will bind via its N-terminal APH domain to the inner leaflet of the plasma membrane. Membrane binding will initiate further oligomerization of SemD which reinforces membrane curvature underneath the invading EB. **b** SemD membrane binding will lead to a local membrane deformation and recruitment of host BAR-domain proteins SNX9 and Pacsin, which will oligomerize and further deform the membrane at the bacterial entry site. In addition, membrane-bound SemD recruits and activates the host actin modulator N-WASP to the invaginating membrane. Both N-WASP and SemD bind G-actin molecules and together with Arp2/3 will induce local actin polymerization. This generates forces (black arrows) which modulate vesicle curvature and diameter to facilitate *Cpn* endocytosis.

The internalization of an obligate intracellular pathogen like *C. pneumoniae* by non-professional phagocytic cells such as epithelial cells is a multifactorial process that involves the hijacking of the host’s receptor-mediated endocytic machinery by specialized pathogen-derived factors. This is necessary to facilitate the efficient uptake of infectious chlamydial EBs with diameters of 300–400 nm, which are much larger than normal endocytic vesicles (~120 nm at most; Fig. 5). Thus, the uptake of EBs requires (i) extensive deformation of nascent pockets in the PM to accommodate each adherent EB, and (ii) control of the local polymerization of branched F-actin to generate sufficient force to enlarge and pinch off the endocytic vesicles to finalize the internalization process.

In this study, we characterized the function of CPn0677, an early secreted *Cpn* effector protein (now named SemD) that is the first known example of an effector protein which can perform both of these crucial tasks. Upon adhesion of the EB to the host cell, the N-terminal amphipathic helix of secreted SemD mediates specific binding to lipids in the inner leaflet of the PM underneath the EB, and may insert into the PM rather like a wedge to induce local membrane bending^35^. Indeed, upon binding to synthetic GUV membranes, SemD transforms them into stable, characteristically angular structures. This membrane deformation is reminiscent of phenotypes observed upon membrane binding by septins or artificially engineered amphipathic DNA nanoparticles. In both of these cases, flat oligomers are formed on the membrane surface and the membrane is bent at a specific angle in the gaps between these arrays^36,37^. Thus, we speculate that, during endocytosis of *Cpn* EBs, secreted SemD binds to and forms oligomers on the inner leaflet of the PM, thus deforming the invaginating pocket, probably in concert with the C-terminal portion of the protein. SemD then recruits the BAR proteins SNX9 and Pacsin (Fig. 5), which further modulate local membrane curvature —with Pacsin acting at the distal edge and SNX9 at the neck of the developing vesicle—in order to accommodate the adherent EB. The need for a sufficient number of SNX9 molecules is met by SemC, another chlamydial membrane-binding protein that recruits SNX9 to the invaginating vesicle^6^.

Intriguingly, membrane-bound SemD has two additional recruitment capacities. It binds to the central actin modulator N-WASP via two CRIB-binding domains, and to G-actin monomers via two WH2 domains. The resulting multiprotein complex is functional, as targeting of the SemD-N-WASP complex to mitochondria leads to the accumulation of F-actin on their outer membranes and induces their disintegration. Similarly, the recruitment of an N-WASP variant, that lacks membrane-binding capacity, to GUVs via membrane-bound SemD in the presence of G-actin and the Arp2/3 complex results in actin polymerization. These experiments indicate that binding of the CRIB domain to the C-terminal domain of SemD overrides the autoinhibition of N-WASP, and enables it to interact with the Arp2/3 complex. This in turn activates the branching activity of the ARP2/3 complex, thus stimulating actin polymerization (Fig. 5). During *Cpn* infection, N-WASP colocalizes with SemD at invading EBs, and chemical inhibition experiments have shown that N-WASP is essential for chlamydial EB uptake. The CRIB-binding motifs in SemD are similar to those found in other mammalian proteins (e.g., Nck) that interact with N-WASP, all of which resemble the C-terminal domain of N-WASP itself^32^. Recruitment and activation of N-WASP via binding to its CRIB domain has also been found in a number of other bacterial effector proteins, such as EspF and EspF(U) in enterohaemorrhagic *Escherichia coli* (EHEC), and the TmeA protein of *C. trachomatis*^33,10^. SemD also harbors two such CRIB-binding domains (CBD), which would enable simultaneous binding of two N-WASP proteins thereby amplifying actin polymerization. Intriguingly and in contrast to other effectors, SemD is the first such effector which performs it recruiting activity while binding and reshaping the plasma membrane.

Our data show that SemD harbors two WH2 domains that specifically bind monomeric G-actin and are present in many eukaryotic actin-binding proteins, but also found in a number of bacterial proteins, where they occur in tandem repeats^38^. SemD is able to bind G-actin not only in pulldown experiments in solution, but also in a membrane-bound form, thus recruiting G-actin to the surface of GUV membranes. While N-WASP contains two WH2 domains that form its V-domain and can therefore bind two actin monomers, the presence of WH2 domains alone is not sufficient for actin polymerization; N-WASP interaction with the Arp2/3 complex brings actin monomers into close proximity, thus facilitating polymerization^38^. During regular endocytosis, activated N-WASP will bind to membrane-bound SNX9 and Pacsin, where it dimerizes^39^. This brings four actin monomers into close proximity to initiate nucleation by the Arp2/3 complex of a new branched actin filament on an existing actin filament at an angle of 70°^40^. These polymerization events generate the forces needed to reshape the endocytic vesicle^7^. During *Cpn* infection, each SemD molecule may deliver additional two actin monomers, thus increasing the local G-actin concentration to promote actin polymerization by increasing the actin nucleation capacity of the N-WASP-Arp2/3 complex and/or the rate of actin polymerization. The SemD-mediated recruitment of SNX9 and Pacsin to the membrane further enhances actin polymerization, as both proteins bind N-WASP via its SH3 domain, thereby also stimulating N-WASP to trigger Arp2/3-dependent polymerization of branched actin filaments.

In conclusion, our data support a model in which the chlamydial effector SemD upon secretion (i) binds and bends the invaginating plasma membrane; (ii) once bound to the membrane, it serves as a recruiting platform for the endocytic proteins SNX9 and Pacsin for further membrane deformation via their BAR domains, and for binding N-WASP for Arp2/3-mediated actin polymerization; (iii) activates N-WASP, while simultaneously binding two G-actin molecules, all of which will maximize the Arp2/3-mediated start of actin branch formation at defined locations in direct contact with the invaginating membrane. Future studies are required to elucidate the mechanistic details as well as the spatial and temporal order of this SemD-driven multifactorial, plasma membrane-bound processes especially how it coordinates its function with SemC, which is present at the same time at the same membranes.

## Methods

### Antibodies and reagents

The primary antibody against SNX9 (OTI1E4, 1:1000) was purchased from Origene, anti-Pacsin (PA5-83983, 1:200), anti-N-WASP (PA5-52198, 1:200) and anti-β-actin (MA5-15739, 1:2000) antibodies were sourced from Thermo Scientific and the anti-actin (#7301-01, 1:500) antibody was from Hypermol. Anti-penta-His (#34660, 1:2500) and anti-GST (#2622, 1:1000) antibodies were obtained from Qiagen and Cell Signaling, respectively. Antibodies against Cpn0677 were generated by Eurogentec (Belgium, 1:50 in immunofluorescence).

Secondary anti-rabbit, anti-rat and anti-mouse antibodies coupled to Alexa488 or Alexa594 (2 µg/ml) or coupled to alkaline phosphatase (1:10,000) were purchased from Thermo Scientific. Rhodamine-Phalloidin (#R415) was purchased from Thermo Scientific. All lipids used in this study were obtained from Avanti Lipids, NHS-FITC and NHS-Rhodamine and MitoTracker™-Red from Thermo Scientific. Wiskostatin (W2270-5MG) was purchased from Merck. G-actin (#8101-01), G-actin-Atto647 (#8158-03) and Arp2/3 (#8413-01) were obtained from Hypermol.

### Plasmid constructs, cloning procedures

For all CPn0677 constructs used in this study, the *cpn0677* gene was amplified from *C. pneumoniae* GiD genomic DNA and integrated into either pSL4 to generate C-terminal 10His-Tag fusions for protein expression or into pKM275 (SNAP) and pKM55 (GFP) for ectopic expression in human cells. GST-Pacsin and mCherry-Pacsin constructs were generated by reverse transcription, followed by amplification of the *PACSIN2* gene (Sequence ID: NM_001184970.3) from total human mRNA isolated from HEp-2 cells. GST‐SNX9_pGEX5.1 was generated by cloning the SNX9 coding sequence into the *BamH*I and *Not*I sites of pGEX5×1^6^. GFP-N-WASP (#47406) and pmCherryC1 Human Wip (#29573) were purchased from Addgene and used as templates for further sub-cloning. Plasmids coding for shRNAs against *PACSIN2* (TRCN0000037979, TRCN0000037980, TRCN0000037981, TRCN0000037982, and TRCN0000037983) were obtained from Merck, and used in combination with psPAX2 and pCMV-VSV-G to generate lentiviral particles for transduction of HEp-2 cells.

### Growth of chlamydia, bacteria, and cell lines

*C. pneumoniae* GiD was propagated in HEp-2 cells (ATCC: CCL-23). Wild-type HEp-2 and HEp-2 cells stably expressing shRNA plasmids were cultured in DMEM medium supplemented with 10% fetal calf serum (FCS), MEM vitamins and non-essential amino acids (Thermo Scientific). Chlamydial elementary bodies (EBs) were purified using a 30% gastrographin solution (Bayer) and stored in SPG buffer (220 mM sucrose, 3.8 mM KH_2_PO_4_, 10.8 mM Na_2_HPO_4_, 4.9 mM l-glutamine). All cloning was carried out by in vivo homologous recombination in *Saccharomyces cerevisiae*. The *Escherichia coli* strains XL-1 Blue (Stratagene) and BL21 (DE3, Invitrogen) were used for plasmid amplification and protein purification, respectively.

### Size-exclusion chromatography

SEC was performed on an ÄKTA™ pure 25 L (Cytivia) using a superdex 200 increase 10/300 GL column (Cytivia) at 4 °C and a flow rate of 0.5 ml/min. 5 mg/ml CPn0677 protein was dissolved in phosphate-buffered saline (PBS) (10 mm Na_2_HPO_4_, 1.8 mm KH_2_PO_4_, 137 mm NaCl, 2.7 mm KCl, pH 6.0) and applied to the column. The void volume of the column was determined by using blue dextran (Merck), and the separation range of the column was verified by standard proteins for gel filtration (MWGF1000-1KT, Merck).

### Co-immunoprecipitation

For in vivo co-immunoprecipitation (Co-IP) of infected cells, HEp-2 cells were first cultivated to 100% confluency in six-well plates and infected with *C. pneumoniae* EBs (MOI 100) that had been gradient-purified by centrifugation for 20 min (25 °C at 2900 rpm). After infection, cells were shifted to 37 °C and grown under 6% CO_2_ for 15 min. The cells were then washed three times with HBSS and lysed with Phospho-Lysis buffer (1% NP40, 1% Triton X100, 20 mM Tris, 140 mM NaCl, 2 mM EDTA, 1 mM Na_2_VO_4_, Roche Protease Inhibitor Cocktail). The lysate was cleared by centrifugation for 10 min at 4 °C at 10,000 × *g*. The supernatant was then mixed with Protein G MicroBeads (Miltenyi Biotech) that had been preincubated with 2 µg anti-SNX9, anti-Pacsin, anti-N-WASP or anti-CPn0677 overnight at 4 °C. Co-IP assays of co-transfected cells were performed using GFP-Trap (Chromotek) following the manufacturer’s protocol. Proteins eluted proteins by either method were subjected to SDS/PAGE and detected by immunoblot analysis using specific antibodies.

### Quantitative mass-spectrometric analysis of CPn0677 co-purified proteins

Proteins isolated by GFP-Trap affinity purification from GFP- and CPn0677-GFP-expressing cells were prepared for mass-spectrometric analysis by in-gel digestion, essentially as described previously^41^. Briefly, proteins were rapidly fractionated in a polyacrylamide gel, stained with Coomassie brilliant blue, reduced with dithiothreitol, alkylated with iodoacetamide, digested with trypsin and finally resuspended in 0.1% trifluoroacetic acid.

The resulting peptides were fractionated on a rapid-separation liquid-chromatography system (Ultimate 3000, Thermo Fisher Scientific) using C18 columns and a 1-h gradient, essentially as described previously^42^. Peptides were directly injected into a QExactive plus mass spectrometer (Thermo Fisher Scientific) using a nano-source interface. The mass spectrometer was operated in positive, data-dependent mode. The following settings were used to record precursor spectra: spectrum resolution: 70,000, advanced gain-control target: 3,000,000, maximum ion time 50 ms, scan range 200 to 2000 *m/z*, profile mode. Up to 20 twofold to fivefold charged precursors were selected (4 *m/z* isolation window), fragmented by higher-energy collisional dissociation and analyzed under the following conditions: spectrum resolution, 17500; advanced gain-control target, 100,000; maximum ion time, 50 ms; available scan range; 200–2000 *m/z*; profile mode. Already fragmented precursors were excluded from further isolation for the next 10 s.

The resulting raw files were further processed using MaxQuant (version 1.6.3.4, Max Planck Institute for Biochemistry, Planegg, Germany) for peptide and protein identification and quantification, with standard parameters unless otherwise stated. Database searches were carried out on the basis of *Homo sapiens* and *C. pneumoniae* entries downloaded from UniProt KB on 20th August 2018 (UP000005640) and additional entries for GFP (Q9Z7M7, Q9Z7M6, P42212). The ‘match between runs’ option, as well as label-free quantification (LFQ), was enabled, methionine oxidation and N-terminal acetylation were considered as variable and carbamidomethylation at cysteines as fixed modification. Peptides and proteins were identified at a false-discovery rate of 1%.

Quantitative data were further processed with Perseus (Version 1.6.2.2, Max Planck Institute for Biochemistry, Planegg, Germany). Only proteins identified based on at least two different peptides and showing at least three valid values in the CPn0677-GFP group were considered for statistical analysis. Here, log_2_ transformed LFQ-intensity values were used. Missing values were filled in with values from a downshifted normal distribution (width 0.3, downshift 2.5 standard deviations) and *P* values calculated by Student’s *t* test, which was combined with the significance-analysis-of-microarrays approach for cutoff determination (S_0_ = 0.2, 10% false-discovery rate).

### Pulldown assays

Recombinant His- or GST-tagged proteins were expressed in *E. coli* BL21 and purified according to the manufacturer’s protocols for cOmplete™ His-Tag purification resin (Merck) and Pierce Glutathione Agarose (Thermo Scientific). Aliquots (100 µg) of purified protein were incubated for 2 h at 4 °C with either His-Tag purification resin or Glutathione Agarose, then 100 µg of the test protein was added and incubated for an additional 2 h at 4 °C. The mixture was washed eight times with ice-cold PBS containing 50 mM imidazole (Merck) or 50 mM TRIS/HCL pH 8 (Merck). Eluted fractions were collected by adding 100 µl of 500 mM imidazole in PBS or 50 mM TRIS/HCl containing 10 mM reduced glutathione. For pulldowns of complexes formed between CPn0677 SNX9 and Pacsin mCherry Trap®, agarose was used. Protein samples were resolved by SDS/PAGE and detected by immunoblot analysis using specific primary and secondary antibodies at 1–2 µg/ml.

### Immunofluorescence staining

Transfected and/or infected cells were fixed at the indicated timepoints with 3% paraformaldehyde in PBS (PFA) for 10 min, then washed three times with PBS, and permeabilized with either 100% methanol for 10 min or with 2% saponin (Merck) in PBS for 20 min. Depending on the permeabilization protocol, primary antibodies were diluted 1–2 µg/ml in PBS or in 0.5% saponin solution, and incubated for 30 min at 37 °C. Cells were washed three times with PBS with or without 0.5% saponin and incubated with secondary antibodies (2 µg/ml, anti-rabbit/ mouse Alexa488/ 594) for 30 min at 37 °C in PBS with or without 0.5% saponin. For detection of SNAP-tagged constructs, transfected cells were incubated for 30 min at 37 °C with 3 µM SNAP-Cell® 647-SiR (New England Biolabs) in medium. Excess dye was removed by washing the cells three times with medium before fixing the cells with PFA and further processing for additional staining procedures depicted above. DAPI was used to visualize DNA.

### Infection/internalization assays

Internalization of EBs was assessed using either HEp-2 cells that stably express shRNA constructs, or HEp-2 cells pretreated for 5 min with DMSO or wiskostatin (10 or 25 µM). For this purpose, cells were cultivated to 70% confluency in 24-well plates on glass coverslips (Ø 1 cm^2^), then infected with purified *C. pneumoniae* EBs (MOI 5) by centrifugation for 20 min (25 °C at 2900 rpm). After centrifugation, cells were shifted to 37 °C and grown under 6% CO_2_ for 2 h, washed three times with PBS, and fixed with 3% paraformaldehyde in PBS (PFA) for 10 min. Internalization rates were determined by immunostaining with anti-LPS (1 µg/ml) and anti-mouse Alexa488 (2 µg/ml) and DAPI. Cells were imaged by confocal microscopy, and internalization ratios were determined by counting external Alexa488-positive and all DAPI-positive bacteria. For the determination of inclusion-body formation, experiments were performed as described above. Cells were washed three times with PBS at 2 hpi, then placed in fresh medium and incubated for an additional 48 h at 37 °C. The numbers of inclusions formed were quantified by confocal imaging, using an antibody directed against the inclusion membrane protein Cpn0147 (1 µg/ml) and anti-rabbit Alexa594 (2 µg/ml), as described previously.

### Preparation and analysis of giant unilamellar vesicles (GUVs)

GUVs were prepared as described previously^6^. Briefly, PIP-containing lipid mixtures were made up of 69.75 mol% DOPC, 25 mol% cholesterol, 0.25 mol% Texas Red™ DHPE, and 5 mol% PIPs. PS-containing GUVs comprised 49.75 mol% DOPC, 25 mol% cholesterol, 0.25 mol% Marina Blue™ DHPE and 25 mol% DOPS. Lipid mixtures were prepared and added to a chamber built of ITO-coated slides (Präzisions Glas & Optik) glued together with Vitrex (Vitrex Medical). The cavity between the slides was filled with 10% sucrose solution and sealed with Vitrex. The slides were connected via clamps to a frequency generator and an alternating voltage of 2.0 Vp-p was applied at a frequency of 11 Hz. The GUVs were grown for 2–3 h in the dark at room temperature.

### Protein binding studies on synthetic GUV membranes

For microscopic analyses, Angiogenesis µ-slides (Ibidi) were coated for 5–10 min at RT with 2 mg/ml beta-casein (Merck) and washed with PBS. Then 10 µl of GUV solution mixed with 30 µl of PBS was added to the slides and the GUVs were allowed to settle. For PIP-binding studies, NHS-FITC-labeled recombinant CPn0677 protein (1 µM) was added and images were acquired at 1-min intervals for 10 min. Images were quantified using ImageJ. For each lipid and protein combination, 50 GUVs were analyzed for the maximum fluorescence intensity on the membrane. For studies of CPn0677 and its binding partners, PS-GUVs labeled with Marina Blue™ were first incubated with the potential binding partner (1 µM) labeled with NHS-rhodamine and imaged for 10 min at RT. Next, 1 µM NHS-FITC-labeled recombinant CPn0677 protein was added, and images were acquired for an additional 10–15 min For actin-binding and actin-polymerization studies Marina Blue™ PS-GUVs were first incubated with 3 µM ice-cold G-actin Atto647 and imaged for 10 min at RT. Then NHS-FITC-labeled recombinant CPn0677 protein (3 µM) was added, and images were acquired for 10–15 min For polymerization studies NHS-FITC-labeled recombinant CPn0677 (3 µM), 100 nM unlabeled Arp2/3 complex, NHS-rhodamine-labeled recombinant mini-WASP (3 µM) and a 1/10^th^ volume of polymerization buffer (500 mM KCl, 20 mM MgCl_2,_ 50 mM guanidine carbonate pH 7.5, 10 mM ATP, 100 mM Tris-HCl, pH 7.5.) and 3 µM ice-cold G-actin Atto647 were added to the GUVs, which were then imaged for 10–15 min.

### Microscopy and image processing

General imaging was performed using an inverse Nikon TiE Live Cell Confocal C2plus equipped with a ×100 TIRF objective and a C2 SH C2 scanner. All images were generated with Nikon NIS Elements software and quantified using ImageJ.

### Statistics and reproducibility

For simple paired analyses between two groups, Student’s *t* test was chosen. A *P* value of less than 0.01 was considered to be statistically significant.

### Reporting summary

Further information on research design is available in the Nature Portfolio Reporting Summary linked to this article.

## Supplementary information

Supplementary Information

Description of Additional Supplementary Files

Supplementary Data 1

Supplementary Movie 1

Supplementary Movie 2

Supplementary Movie 3

Supplementary Movie 4

Reporting Summary

## Data Availability

All data discussed in the paper will be made available to the readers. We included all source data to Figs. 1b, e, 2g, h, 3b into Supplementary Data_1. All uncropped and unedited immunoblots shown in Figs. 2–4, S1, S2 are shown as Supplementary Fig. S3 and can be found in the Supplementary Information file. Source data to Fig. 2a is deposited and accessible in the ProteomeXchange Consortium via the PRIDE^43^ partner repository with the dataset identifier PXD041847.
