# Supplementary material for: A single chlamydial protein reshapes the plasma membrane and serves as recruiting platform for central endocytic effector proteins
Source: Commun Biol. 2023 May 13;6:520. doi: 10.1038/s42003-023-04913-z (PMC10182996; doi:10.1038/s42003-023-04913-z)

Supplementary Information

A single chlamydial protein reshapes the plasma membrane and serves as recruiting platform for central endocytic effector proteins

Dominik Spona[1], Phillipp T. Hanisch[1], Johannes H. Hegemann[1,*], Katja Mölleken[1,*,#]

[1] Institute for Functional Microbial Genomics, Heinrich-Heine-University, Düsseldorf, Germany

[*] Joint senior authors

[#] Corresponding author katja.moelleken@hhu.de

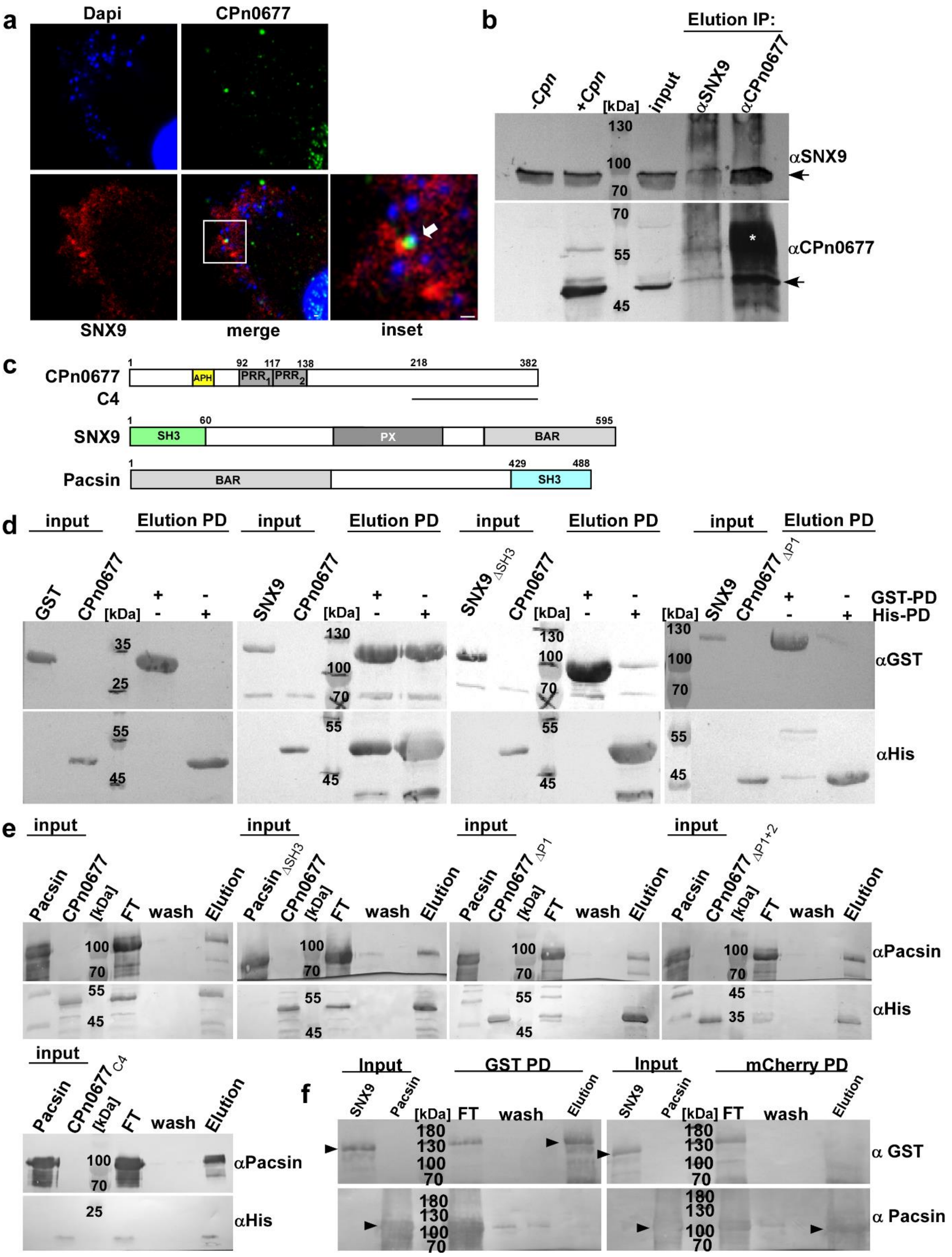

Supplementary Figure S1: CPn0677 interacts with the BAR proteins Pacsin and SNX9 via different protein domains.

(a) Colocalization of SNX9 (stained with anti-SNX9 and anti-mouse Alexa594) and CPn0677 (stained with anti-CPn0677 and anti-rat Alexa488) at bacterial entry sites at 15 min pi. *C. pneumoniae* EBs were stained with DAPI. The inset shows the region outlined by the white frame. The white arrowhead shows colocalization. Bar = 10 µm, inset = 1 µm. **(b)** Co-immunoprecipitation of HEp-2 cells infected (or not) with *C. pneumoniae* EBs (MOI 100). Cell lysates were incubated with µMACS protein G microbeads coupled to antibodies directed against either SNX9 or CPn0677. Eluates were fractionated by SDS/PAGE and incubated with the appropriate antibodies. White asterisks mark the heavy chain of the antibody, the black arrows indicate the specifically labeled SNX9 and CPn0677 bands. **(c)** Schematic representation of the proteins and their known domains used in the pulldown experiments (D,E). Based on these schemes truncated variants used in (D,E) of all proteins were generated. **(d, e)** Pulldown experiments using recombinant full-length or truncated versions of $CPn0677_{10His}$ in combination with the recombinant BAR domain proteins SNX9 (D) and Pacsin fused to GST (E). Input and elution samples obtained from His-pulldowns and GST-pulldowns were fractionated by SDS/PAGE and probed with anti-GST and anti-His antibodies. **(d)** GST, GST-SNX9 and GST-SNX9ΔSH3 were tested for interaction with $CPn0677_{10His}$ and $CPn0677ΔP1_{10His}$ in His-pulldowns and GST-pulldowns. **(e)** GST-Pacsin and GST-PacsinΔSH3 were tested against $CPn0677_{10His}$, $CPn0677ΔP1_{10His}$ $CPn0677ΔP1+P2_{10His}$, and $CPn0677\text{-}C4_{10His}$ in His-pulldowns. **(f)** Control experiment referring to Fig.2 E,F of mixed SNX9-GST and Pacsin-mCherry protein alone either purified via GST or mCherry Trap® agarose. Fractions from both pulldowns were separated by SDS/PAGE and probed with anti-GST, anti-Pacsin antibodies. Black arrowheads indicate full length protein in input and final elution fractions.

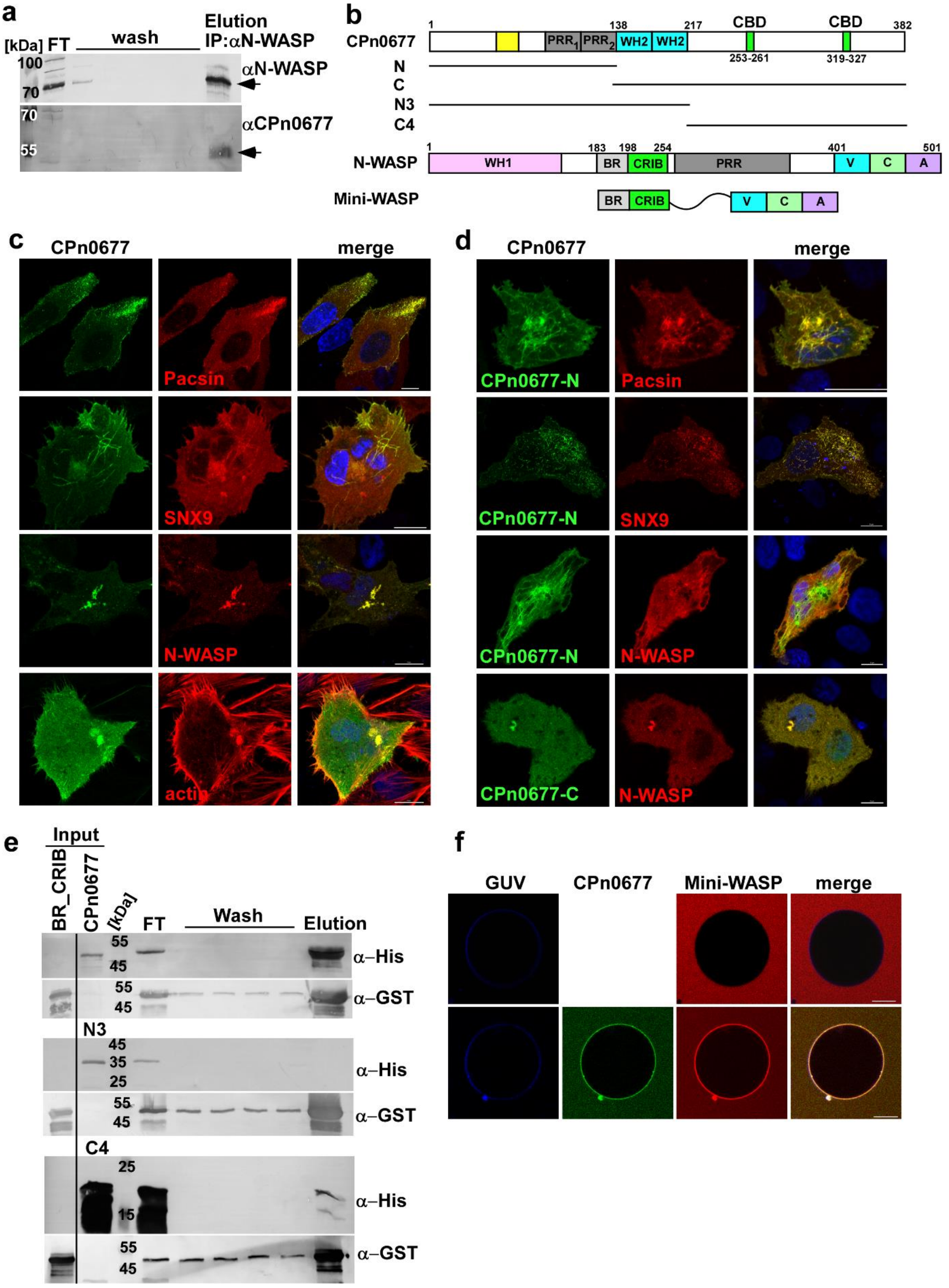

Supplementary Figure S2: CPn0677 and N-Wasp interaction is essential for *Cpn* endocytosis.

(a) Co-immunoprecipitation of HEp-2 cells infected (or not) with *C. pneumoniae* (MOI 100) for 15 min. Cell lysates were incubated with µMACS protein G microbeads coupled to antibodies directed against N-WASP. Elution samples were fractionated by SDS/PAGE and detected with antibodies specific for N-WASP and CPn0677. Black arrows mark the specifically labeled N-WASP and CPn0677 bands. **(b)** Schematic representation of the domain structures of N-WASP (https://www.uniprot.org/uniprot/O00401) and CPn0677 with the predicted WH2 domains (turquoise box, http://elm.eu.org/) and the predicted CRIB-binding motifs (green boxes, http://elm.eu.org/). Sub-fragments of both proteins used in this study are depicted. **(c, d)** Confocal images of HEp-2 cells co-expressing wild-type **(c)** or truncated variants of CPn0677 **(d)** fused to GFP together with Pacsin, SNX9 or N-WASP fused to mCherry. **(c)** Cells expressing CPn0677 alone were fixed and stained for F-actin using rhodamine-phalloidin. DNA was visualized with DAPI. Bar = 10 µm. **(e)** Pulldown experiments using His-tagged CPn0677, CPn0677-N3 or CPn0677-C4 in combination with BR_CRIB fused to GST. Input, flow through (FT), wash and elution samples obtained from His-pulldowns were fractionated by SDS/PAGE and probed with anti-GST and anti-His antibodies. **(f)** Confocal images of PS-GUVs labeled with Marina Blue™ and incubated and imaged for 10 min with NHS-rhodamine-labeled mini-WASP alone (top row), followed by the addition of FITC-labeled CPn0677 (bottom row). See movies S3 6-7 in the Supplementary Information. Bar = 10 µm.

Supplementary Figure 3: Uncropped and unedited immunoblots shown in Figures 2-4, S1, S2.

Spona et al: Original Immunoblots to Figure 2C

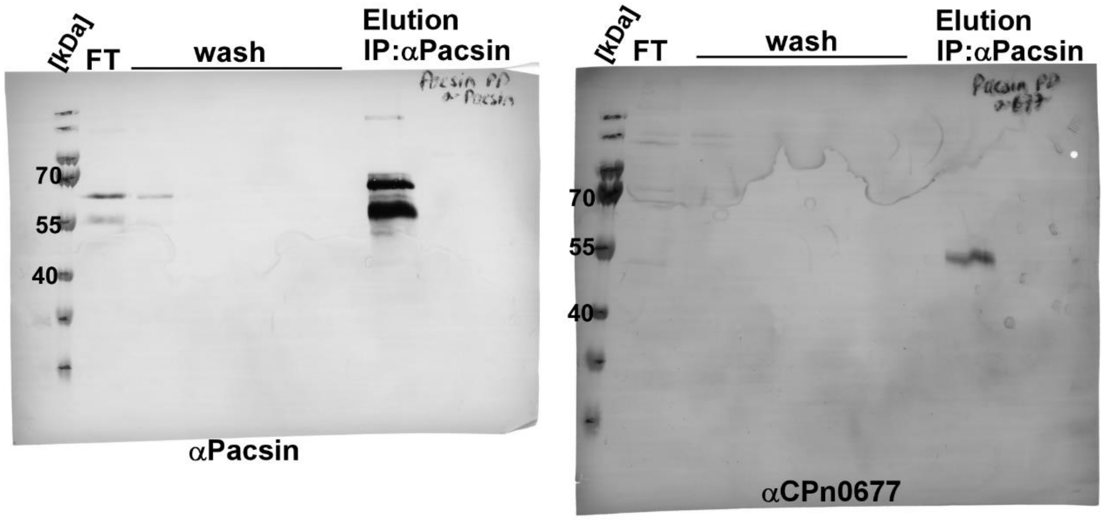

Spona et al: Original Immunoblots to Figure 2I

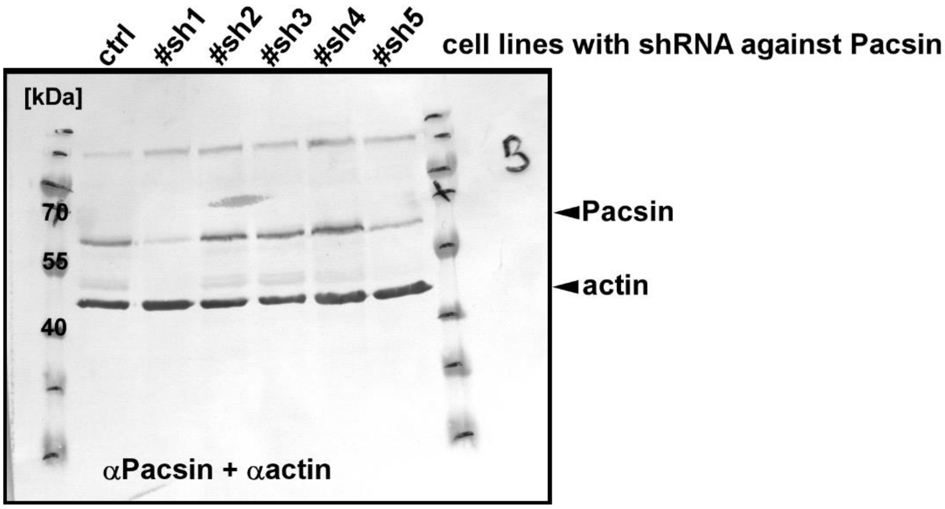

Spona et al: Original Immunoblots to Figure 2E, 2F

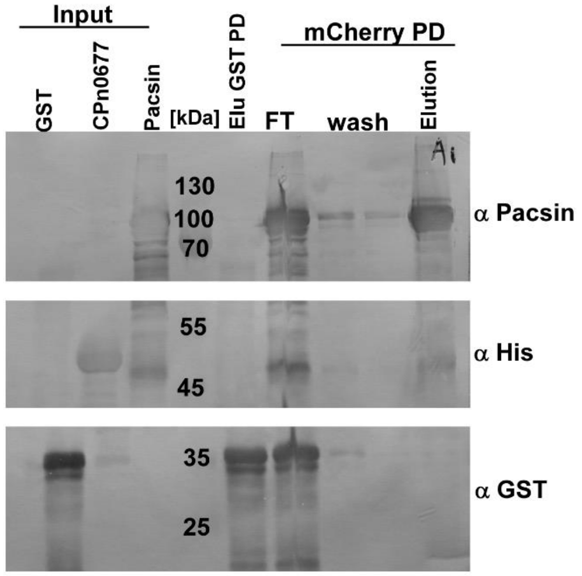

GST + CPn0677 --> GST PD + PacsinmCherry --> mCherry Trap

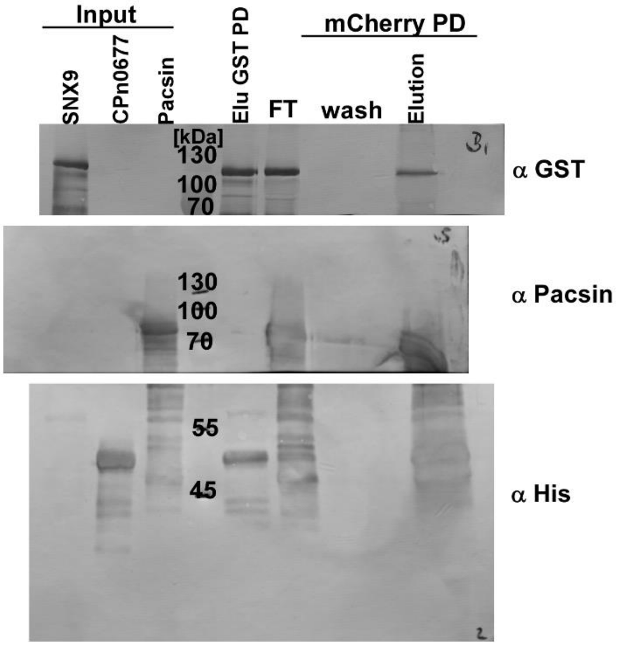

SNX9GST + CPn0677 --> GST PD + PacsinmCherry --> mCherry Trap

Spona et al: Original Immunoblots to Figure S1F

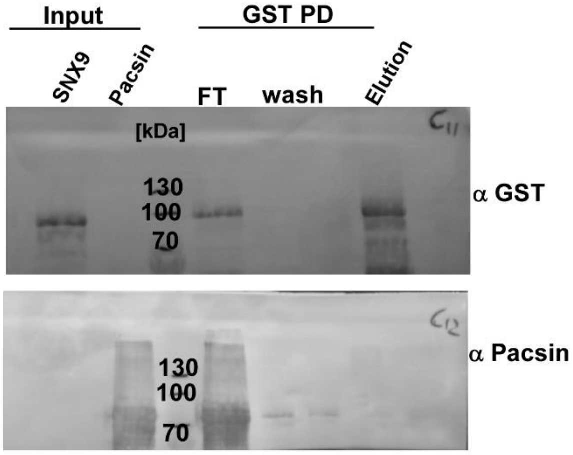

SNX9GST + PacsinmCherry --> GST PD

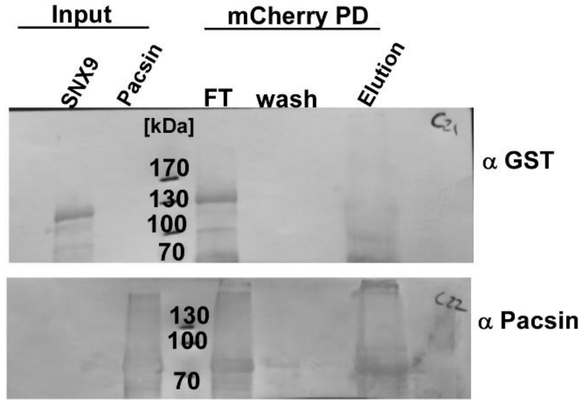

SNX9GST + PacsinmCherry --> mCherry Trap

Spona et al: Original Immunoblots to Figure 4A

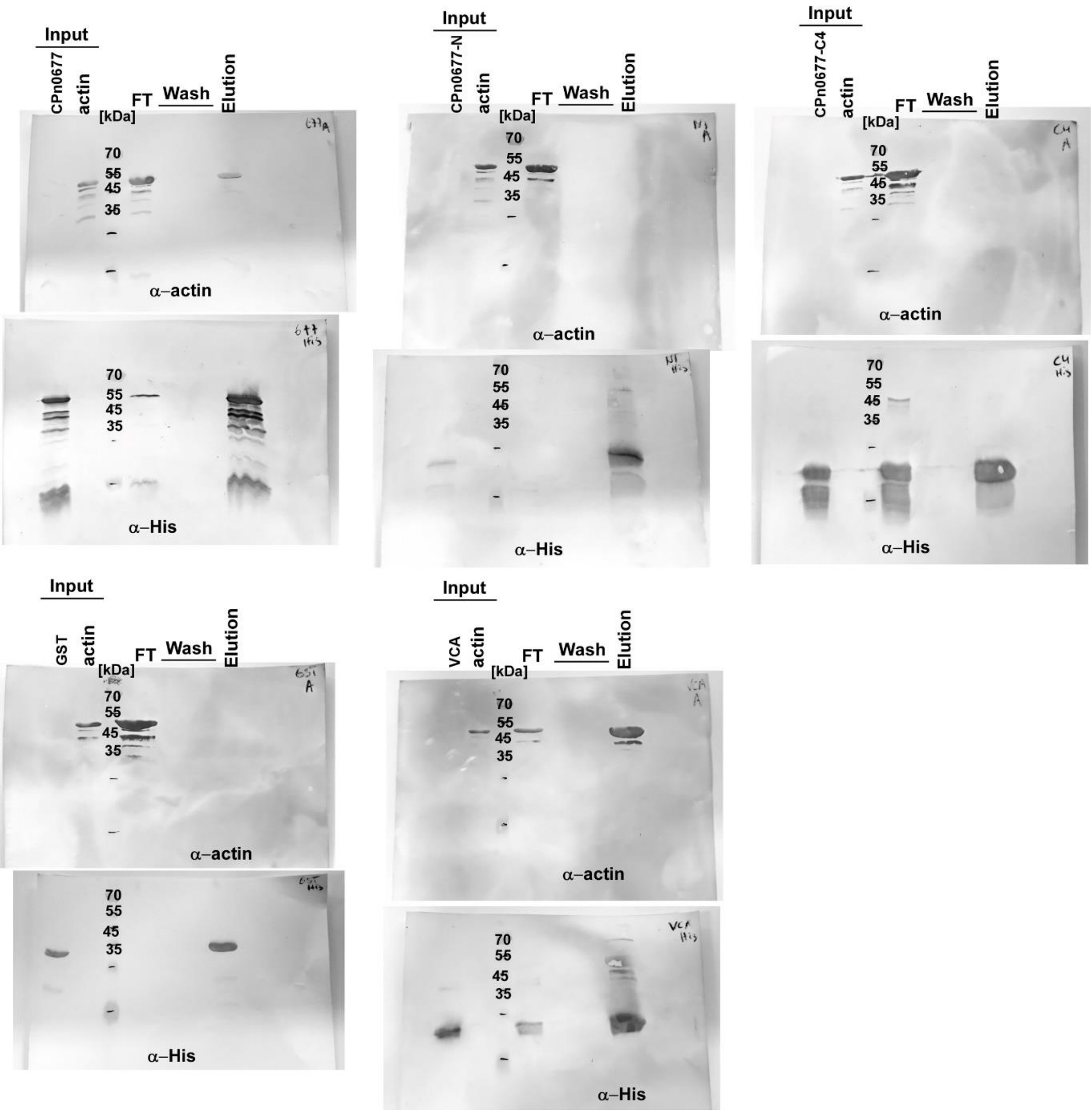

Spona et al: Original Immunoblots to Figure S1B

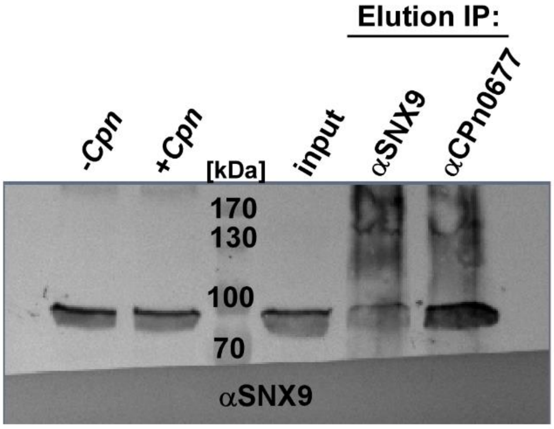

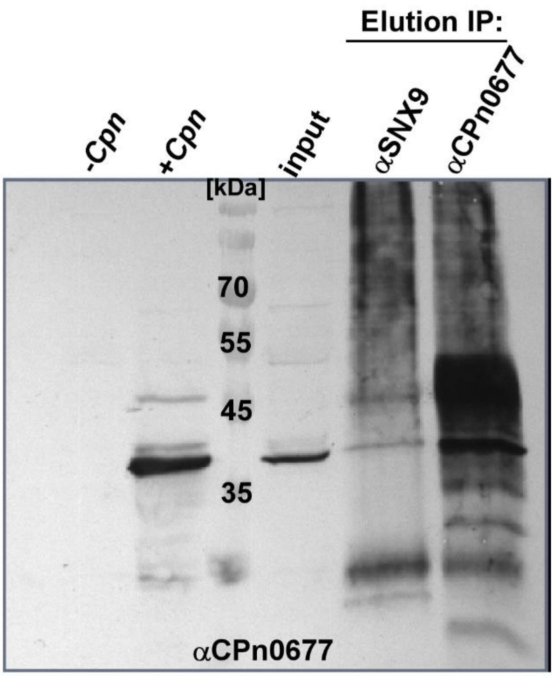

Spona et al: Original Immunoblots to Figure S1D

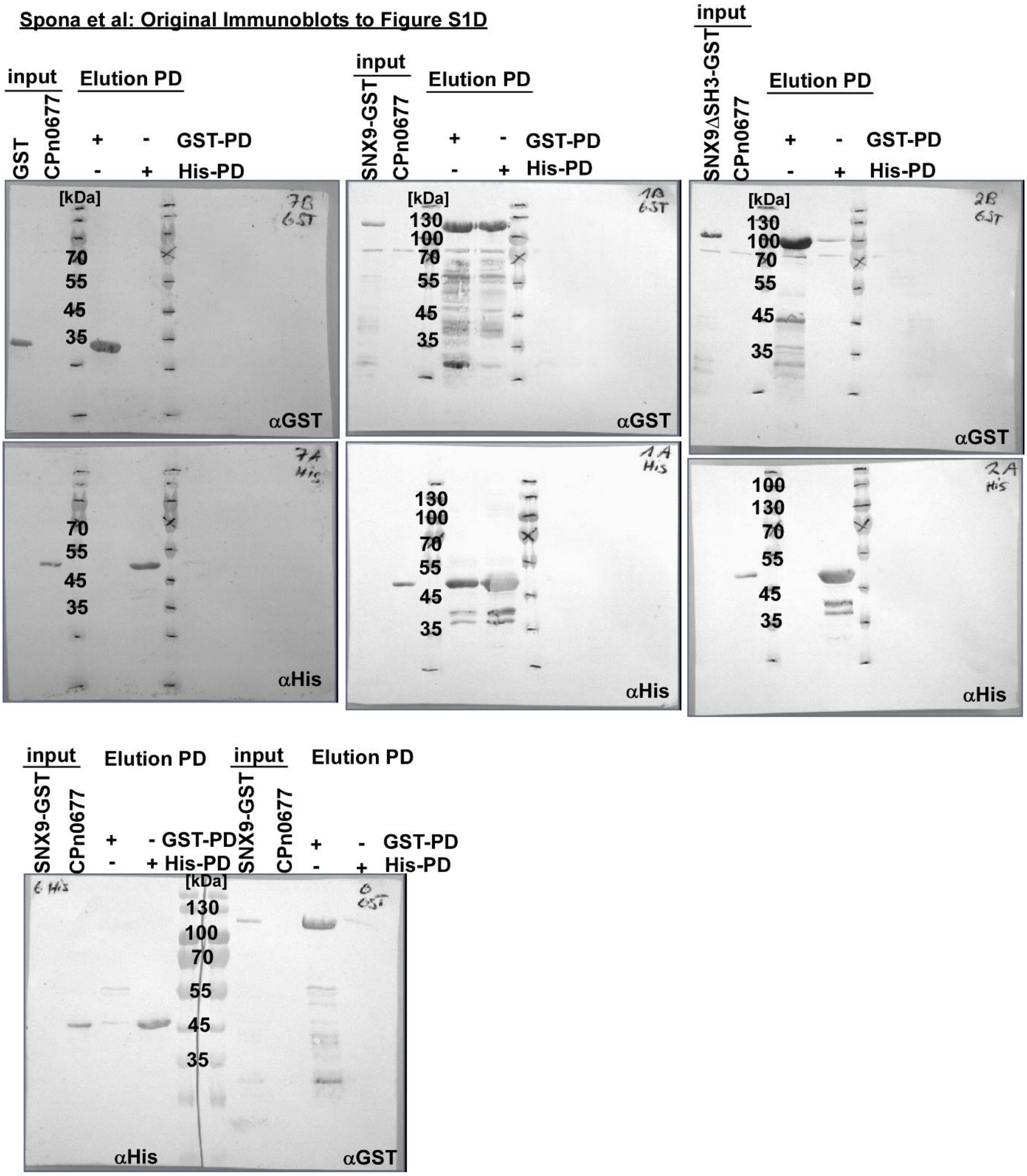

Spona et al: Original Immunoblots to Figure S1E

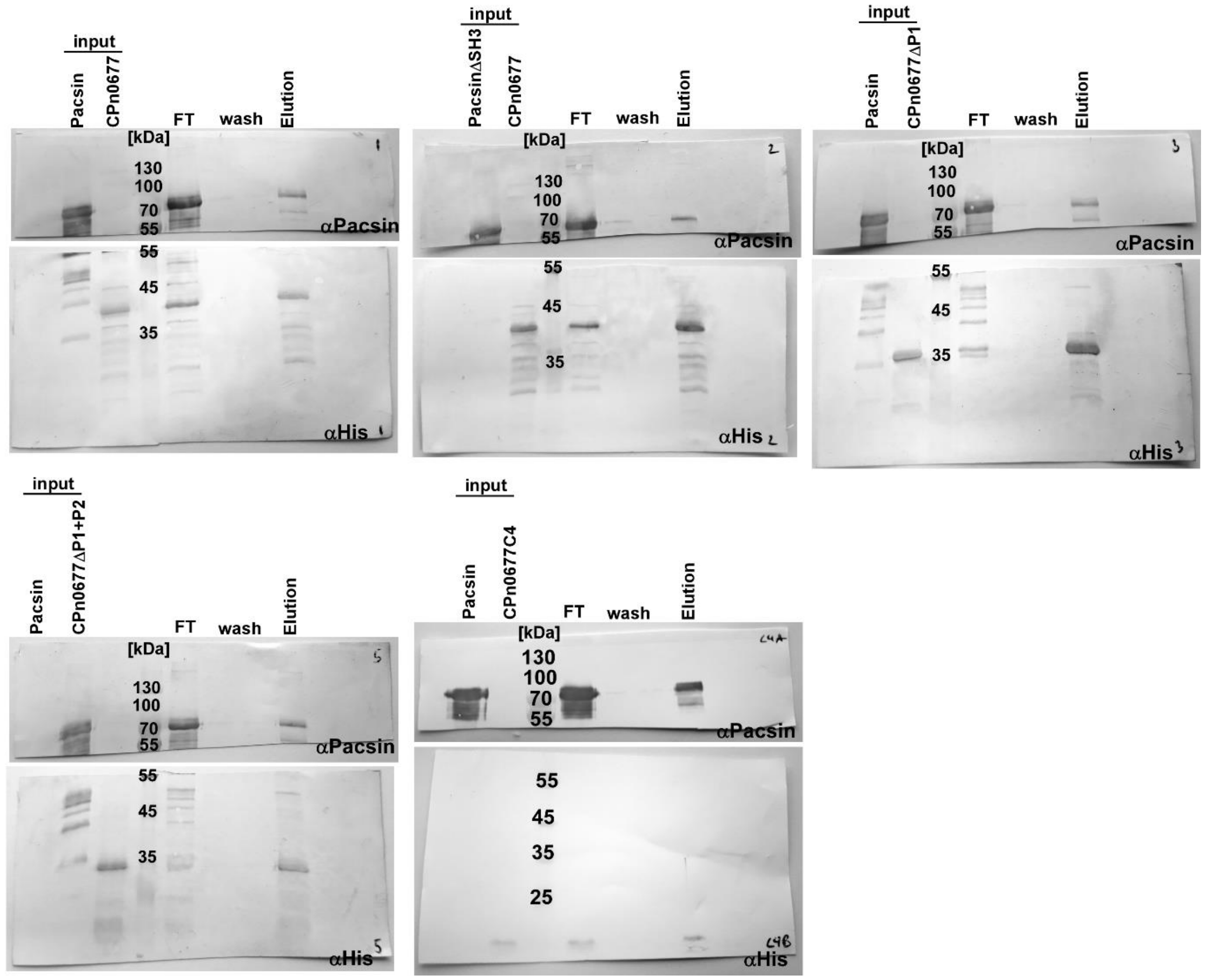

Spona et al: Original Immunoblots to Figure S2A

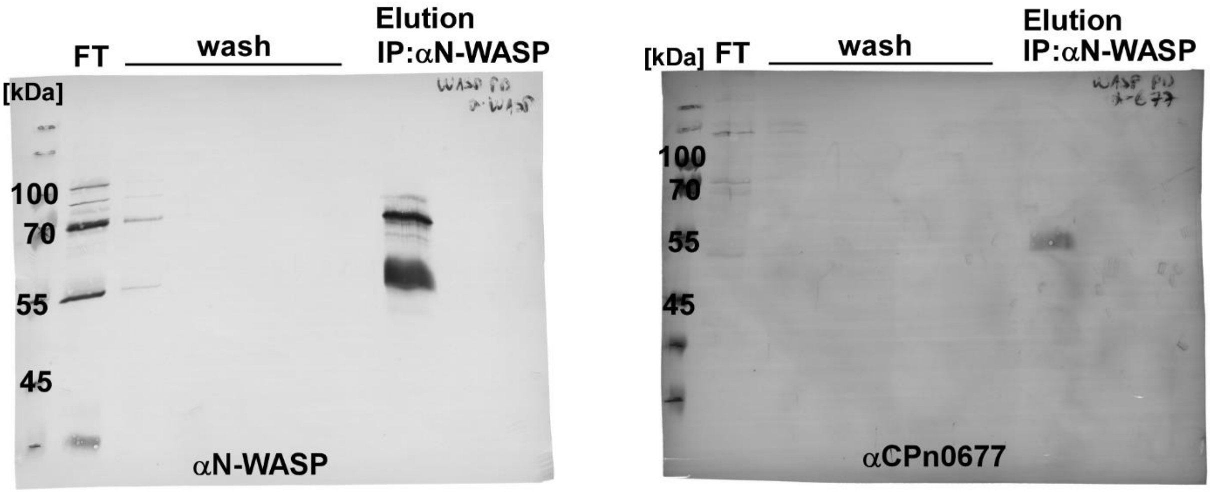

Spona et al: Original Immunoblots to Figure S2E

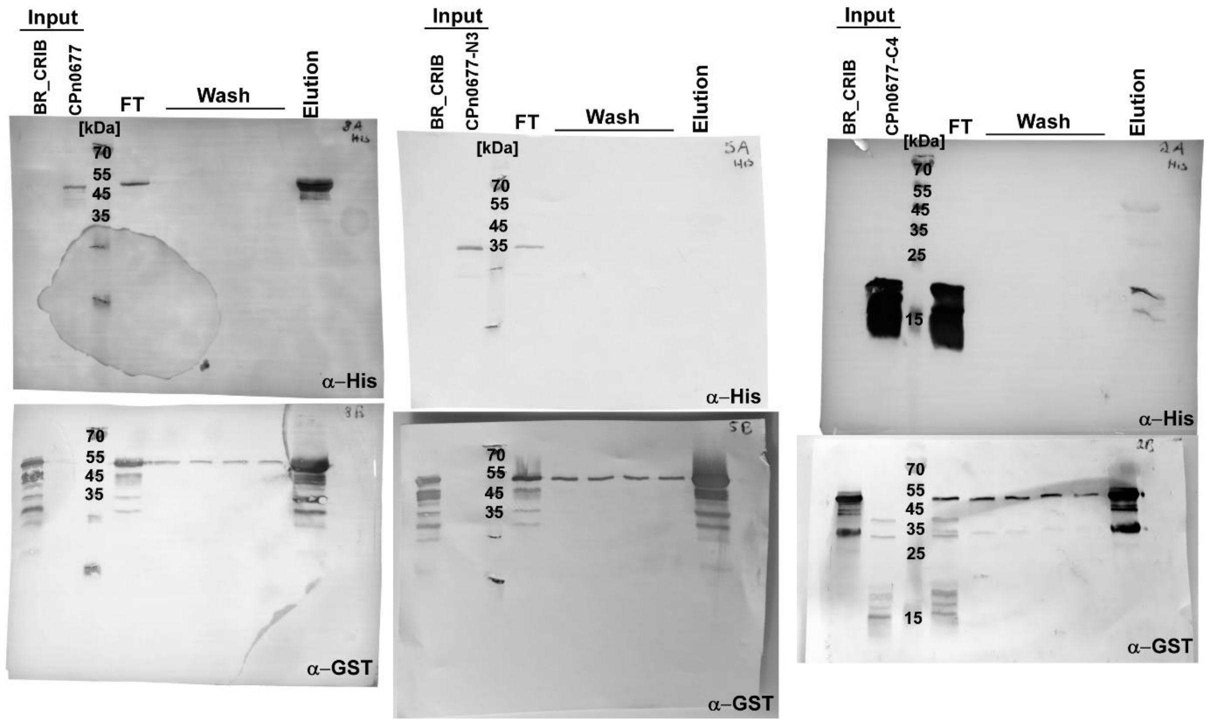

Supplement: Supplementary file 1 — Supplementary Information [file 42003_2023_4913_MOESM1_ESM.pdf]
