# Supplementary material for: A single chlamydial protein reshapes the plasma membrane and serves as recruiting platform for central endocytic effector proteins
Source: Commun Biol. 2023 May 13;6:520. doi: 10.1038/s42003-023-04913-z (PMC10182996; doi:10.1038/s42003-023-04913-z)
Supplement: Supplementary file 2 — Description of Additional Supplementary Files [file 42003_2023_4913_MOESM2_ESM.pdf]

Description of Additional Supplementary Files

File name: Supplementary Data 1
Description: Source data to Figures 1b, 1e, 2g, 2h, 3b.

File name: Supplementary Movie 1
Description: PS-GUVs labeled with Marina Blue™ were incubated with FITC-labeled recombinant CPn0677 (1 µM) and imaged for 15 min. Images were acquired every 1 min.

File name: Supplementary Movie 2
Description: CPn0677 recruits G-actin to GUV membranes. PS-GUVs labeled with Marina Blue™ were incubated with 3 µM G-actinAtto647 for 10 min. Then 3 µM FITC-labeled CPn0677 was added and images were acquired at 1-min intervals for 10 min every 1 min.

File name: Supplementary Movie 3
Description: PS-GUVs labeled with Marina Blue™ were incubated with 3 µM G-actinAtto647, 3 µM NHS-rhodamine-labeled mini-WASP and the unlabeled Arp2/3 complex (100 nM) for 10 min. Images were acquired at 1-min intervals.

File name: Supplementary Movie 4
Description: PS-GUVs labeled with Marina Blue™ were incubated with 3 µM G-actinAtto647 for 10 min. Then 3 µM NHS-rhodamine-labeled mini-WASP, 100 nM unlabeled Arp2/3 complex, 3 µM FITC-labeled CPn0677, and 1x polymerization buffer were added and images were acquired at 1-min intervals for 10-15 min.
