# Supplementary material for: A single chlamydial protein reshapes the plasma membrane and serves as recruiting platform for central endocytic effector proteins
Source: Commun Biol. 2023 May 13;6:520. doi: 10.1038/s42003-023-04913-z (PMC10182996; doi:10.1038/s42003-023-04913-z)
Supplement: Supplementary file 8 — Reporting Summary [file 42003_2023_4913_MOESM8_ESM.pdf]

nature portfolio

Corresponding author(s): Katja Mölleken

Last updated by author(s): Apr 28, 2023

Reporting Summary

Nature Portfolio wishes to improve the reproducibility of the work that we publish. This form provides structure for consistency and transparency in reporting. For further information on Nature Portfolio policies, see our Editorial Policies and the Editorial Policy Checklist.

Statistics

For all statistical analyses, confirm that the following items are present in the figure legend, table legend, main text, or Methods section.

n/a	Confirmed	
☐	☒	The exact sample size (*n*) for each experimental group/condition, given as a discrete number and unit of measurement
☐	☒	A statement on whether measurements were taken from distinct samples or whether the same sample was measured repeatedly
☐	☒	The statistical test(s) used AND whether they are one- or two-sided<br>*Only common tests should be described solely by name; describe more complex techniques in the Methods section.*
☒	☐	A description of all covariates tested
☒	☐	A description of any assumptions or corrections, such as tests of normality and adjustment for multiple comparisons
☐	☒	A full description of the statistical parameters including central tendency (e.g. means) or other basic estimates (e.g. regression coefficient) AND variation (e.g. standard deviation) or associated estimates of uncertainty (e.g. confidence intervals)
☐	☒	For null hypothesis testing, the test statistic (e.g. *F*, *t*, *r*) with confidence intervals, effect sizes, degrees of freedom and *P* value noted<br>*Give P values as exact values whenever suitable.*
☒	☐	For Bayesian analysis, information on the choice of priors and Markov chain Monte Carlo settings
☒	☐	For hierarchical and complex designs, identification of the appropriate level for tests and full reporting of outcomes
☒	☐	Estimates of effect sizes (e.g. Cohen's *d*, Pearson's *r*), indicating how they were calculated

Our web collection on statistics for biologists contains articles on many of the points above.

Software and code

Policy information about availability of computer code

Data collection	1. All confocal imaging data was obtained using a Nikon TiE Live Cell Confocal C2plus equipped with a 100x TIRF objective and a C2 SH C2 scanner.<br>2. Mass spectrometry data were optaines from a QExactive plus mass spectrometer (Thermo Fisher Scientific) using a nano-source interface.
Data analysis	1. All confocal imaging data was Nikon NIS Elements software and quantified using ImageJ.<br>2. Mass spectrometry data was further processed using MaxQuant (version 1.6.3.4, Max Planck Institute for Biochemistry, Planegg, Germany).

For manuscripts utilizing custom algorithms or software that are central to the research but not yet described in published literature, software must be made available to editors and reviewers. We strongly encourage code deposition in a community repository (e.g. GitHub). See the Nature Portfolio guidelines for submitting code & software for further information.

Data

Policy information about availability of data

All manuscripts must include a data availability statement. This statement should provide the following information, where applicable:

- Accession codes, unique identifiers, or web links for publicly available datasets
- A description of any restrictions on data availability
- For clinical datasets or third party data, please ensure that the statement adheres to our policy

All data discussed in the paper will be made available to the readers. We included all source data to Figures 1b, 1e, 2g, 2h, 3b into Supplementary Data_1.

Supplementary Data_2 contains all uncropped and unedited immunoblots shown in Figures 2-4, S1, S2. Source data to Fig. 2a is deposited and accessible in the ProteomeXchange Consortium via the PRIDE [43] partner repository with the dataset identifier PXD041847.

Human research participants

Policy information about studies involving human research participants and Sex and Gender in Research.

Reporting on sex and gender	NA
Population characteristics	NA
Recruitment	NA
Ethics oversight	NA

Note that full information on the approval of the study protocol must also be provided in the manuscript.

Field-specific reporting

Please select the one below that is the best fit for your research. If you are not sure, read the appropriate sections before making your selection.

☒ Life sciences ☐ Behavioural & social sciences ☐ Ecological, evolutionary & environmental sciences

For a reference copy of the document with all sections, see nature.com/documents/nr-reporting-summary-flat.pdf

Life sciences study design

All studies must disclose on these points even when the disclosure is negative.

Sample size	No Sample size was calculated in advance. Sample sizes were choosen to represent results of the biological replicates in each experimental setting.
Data exclusions	No data was excluded from the analyses.
Replication	The experiments were carried out in at least 3 different biological replicates
Randomization	Randomization is a given in our biological replicates settings. Each experiment is performed with fresh and newly prepared ingrediants. E.g. cells are seeded from different stocks on different days. Bacteria are taken from different stock sample. For the GUV analyses, GUV and protein preparations are prepared fresh for each individual experiment.
Blinding	Blinding was not applied during the experiments.

Reporting for specific materials, systems and methods

We require information from authors about some types of materials, experimental systems and methods used in many studies. Here, indicate whether each material, system or method listed is relevant to your study. If you are not sure if a list item applies to your research, read the appropriate section before selecting a response.

Materials & experimental systems

n/a	Involved in the study	
☐	☒	Antibodies
☐	☒	Eukaryotic cell lines
☒	☐	Palaeontology and archaeology
☒	☐	Animals and other organisms
☒	☐	Clinical data
☒	☐	Dual use research of concern

Methods

n/a	Involved in the study	
☒	☐	ChIP-seq
☒	☐	Flow cytometry
☒	☐	MRI-based neuroimaging

Antibodies

Antibodies used	The primary antibody against SNX9 (OTI1E4, 1:1000) was purchased from Origene, anti-Pacsin (PA5-83983, 1:200), anti-N-WASP (PA5-52198, 1:200) and anti-β-actin (MA5-15739, 1:2000) antibodies were sourced from Thermo Scientific and the anti-actin (#7301-01, 1:500) antibody was from Hypermol. Anti-penta-His (#34660, 1:2500) and anti-GST (#2622, 1:1000) antibodies were

obtained from Qiagen and Cell Signaling, respectively. Antibodies against Cpn0677 were generated by Eurogentec (Belgium, 1:50 in immunofluoresce).
Secondary anti-rabbit, anti-rat and anti-mouse antibodies coupled to Alexa 488 or Alexa594 (2µg/ml) or coupled to alkaline phosphatase (1:10000) were purchased from Thermo Scientific.

Validation

Antibodies were used according manufacturers protocols.

Eukaryotic cell lines

Policy information about cell lines and Sex and Gender in Research

Cell line source(s)	HEp-2 Cells, ATCC CCL-23, male
Authentication	HEp-2 cells were non authenticated.
Mycoplasma contamination	Cells are Mycoplasma-negative tested by PCR.
Commonly misidentified lines (See ICLAC register)	NA
